# Noise reduction facilitated by dosage compensation in gene networks

**DOI:** 10.1038/ncomms12959

**Published:** 2016-10-03

**Authors:** Weilin Peng, Ruijie Song, Murat Acar

**Affiliations:** 1 Department of Molecular, Cellular and Developmental Biology, Yale University, 219 Prospect Street, New Haven, Connecticut 06511, USA; 2 Systems Biology Institute, Yale University, 850 West Campus Drive, West Haven, Connecticut 06516, USA; 3 Interdepartmental Program in Computational Biology and Bioinformatics, Yale University, 300 George Street, Suite 501, New Haven, Connecticut 06511, USA; 4 Department of Physics, Yale University, 217 Prospect Street, New Haven, Connecticut 06511, USA

## Abstract

Genetic noise together with genome duplication and volume changes during cell cycle are significant contributors to cell-to-cell heterogeneity. How can cells buffer the effects of these unavoidable epigenetic and genetic variations on phenotypes that are sensitive to such variations? Here we show that a simple network motif that is essential for network-dosage compensation can reduce the effects of extrinsic noise on the network output. Using natural and synthetic gene networks with and without the network motif, we measure gene network activity in single yeast cells and find that the activity of the compensated network is significantly lower in noise compared with the non-compensated network. A mathematical analysis provides intuitive insights into these results and a novel stochastic model tracking cell-volume and cell-cycle predicts the experimental results. Our work implies that noise is a selectable trait tunable by evolution.

## Abstract (web summary)

Cells must function despite the noisiness of their processes by tolerating or reducing such variability. Here, the authors combine experiment and modelling to show that a network motif that mediates network-dosage compensation also reduces noise in network output, suggesting that noise is tuneable.

## Graphical abstract

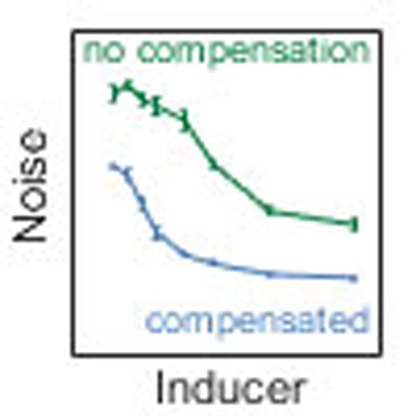

The dosage of a gene network, defined as the number of copies of the network in a cell, naturally changes throughout the cell cycle due to chromosome duplication events. Further, a variety of other effects, such as global variations in gene expression and changes in cell volume, affect all genes in a gene network and so can be thought of as changes in effective network dosage^1^,^2^. Unless the cell utilizes network-dosage compensation strategies, such dosage changes can be detrimental to cellular phenotypes. Network-dosage compensation refers to the phenomenon in which the output of a gene network is invariant to changes in network dosage; this is different from gene dosage compensation^3^, which is about changes in the copy number of individual genes rather than entire gene networks. A previous study^4^ has shown that the output or activity of a gene network could be invariant to alterations in network-dosage via a molecular mechanism intrinsic to the network structure. The mechanism can operate in any network containing at least two regulatory components, one positive and one negative regulator. These two components have to interact with a 1-to-1 stoichiometry under specific network topologies allowing only one of them to directly affect network activity. A subsequent genome-wide study^5^ has shown that approximately one-third of the yeast gene networks analysed satisfied the requirements for network-dosage compensation, indicating that the property might have been selected over evolutionary time scales.

The activity of a natural gene network can display varying degrees of fluctuations or noise^2^,^6^,^7^,^8^,^9^ even in genetically identical cells or in a single cell over time^10^. Extrinsic noise, which is caused by cell-to-cell variations in global factors such as the number of ribosomes in a cell or the cell volume, contributes significantly to the overall noise in the activity of many gene networks^2^,^7^,^8^,^9^,^11^. As many sources of extrinsic noise (for example, changes in cell volume, or variations in the abundance of ribosomes, general transcription factors or RNA polymerase) equally affect all genes in the network, noise from those sources may be thought of as effectively altering the dosage level of the gene network, though more fine-grained than actual changes in the network copy number. Indeed, the mathematical model of network-dosage compensation in the previous work^4^ has no dependency on network-dosage changes being in discrete, coarse-grained steps and even coarse-grained changes in network dosage will result in much finer-grained changes downstream in different cells, owing to the variation in transcription, translation, growth and degradation rates. On the basis of a mathematical analysis (Supplementary Note 1), it is reasonable to expect that the activity of a dosage-compensated network would be less sensitive to such noise sources compared with non-compensated networks, thus reducing the effects of a significant contributor to overall noise level. We therefore hypothesize that the activity of dosage compensated networks is less noisy compared with networks that are not compensated. To test this hypothesis, we use the canonical galactose (GAL) network^4^,^12^,^13^,^14^ as experimental model in the yeast *Saccharomyces cerevisiae*.

The GAL network has a well-characterized^4^,^12^,^13^,^14^ bistable^15^,^16^,^17^,^18^ activity, which has previously been shown to be network-dosage compensated^4^. More specifically, it was shown that the network activity, defined as the fraction of induced cells occupying the ON state, was similar between networks carrying one or two copies of the network genes. The specific interaction topology connecting *GAL3* and *GAL80* to *GAL4* was a requirement for the compensated phenotype.

In the GAL network, a small set of regulatory genes takes key roles^4^,^12^,^19^,^20^,^21^,^22^ in regulating network activity. Galactose molecules are imported into the cell by hexose transporters, including the non-essential^19^ *GAL2* permease. *GAL4* is a constitutively expressed transcriptional activator regulating the expression of all other genes in the network by binding to their promoters. *GAL80* is a repressor which prevents transcription when bound to *GAL4*. *GAL3* carries inducer function in the network. The galactokinase *GAL1* is highly homologous to *GAL3*, and is also capable of inducing the expression of the network genes^23^,^24^, although its inducer activity is about 40-fold weaker than *GAL3*. Galactose molecules activate the inducer proteins and the activated proteins can bind to *GAL80*, relieving *GAL4* from the inhibitory effect of *GAL80* and therefore promoting transcription. Mediated by proteins that function as inducers and repressors in the network, key positive and negative feedback loops are embedded into the network^4^,^12^,^14^.

Here, we synthetically modify the GAL network to abolish the network-dosage compensation phenotype, which results in a significant increase in the level of noise. A cell-volume- and cell-cycle-aware stochastic model predicts the experimental results. Our results suggest that network-dosage compensation can operate to reduce the effects of extrinsic noise.

## Results

### Quantifying the activity of the native GAL network

**Figure 1:**
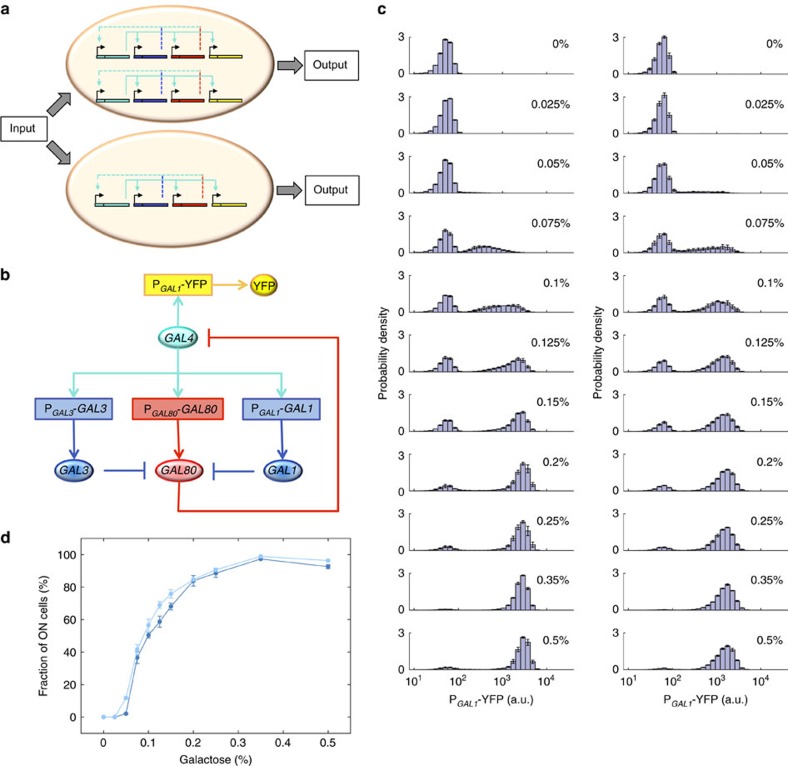
Topology and compensated response of the natural network. (**a**) Cells with one or two copies of the natural GAL network. For each copy of the network, key network components (*GAL4, GAL3 & GAL1, GAL80* and P_*GAL1*_-YFP) are represented by turquoise, blue, red and yellow, respectively. The regulation by *GAL4* is indicated by solid turquoise lines and feedback loops are shown by dashed lines. (**b**) Natural network architecture built by key regulatory components. Coloured ellipses depict proteins, while rectangles show the promoter-gene regions on DNA. Pointed turquoise and blue/red arrows indicate activation and gene expression, respectively, while blunt arrows reflect inhibition. (**c**) At different galactose concentrations, single-cell expression histograms show the response of diploid *S. cerevisae* strains carrying the natural GAL network topology (a.u., arbitrary units). At left, diploid strain WP128 with two copies of the natural network; at right, diploid strain WP232 with one copy of the natural network. (**d**) Fraction of ON cells as a function of galactose concentration from diploid cells carrying one (WP232, light blue) or two (WP128, dark blue) copies of the natural network. Error bars indicate s.e.m. (*N*=4).

To quantify the activity of the GAL network (Fig. 1a,b) at the single-cell level, we used the yellow fluorescent protein (YFP) driven by the *GAL1* promoter as our reporter. The activity of the *GAL1* promoter is a reliable representative of the network activity, as this promoter has *GAL4* binding sites. We induced the network at eleven different galactose levels for 22 h in diploid cells and measured single-cell expression profiles by flow cytometry (Supplementary Note 2, Supplementary Figs 1–3). The network activity displayed a bimodal profile with the fraction of ON cells increasing with increasing galactose concentration (Fig. 1c, left column). Next, to observe the network level dosage compensation in the GAL network, we deleted one of the two copies of *GAL2, GAL3, GAL1, GAL4* and *GAL80* in diploid background and measured the activity of the heterozygous network at several galactose induction levels (Fig. 1c, right column). We found the network activity to be invariant (Fig. 1d, Supplementary Note 3) to halving the dosage of the natural network, reaffirming the results of the previous work^4^.

### Synthetic modification abolishes network-dosage compensation

**Figure 2:**
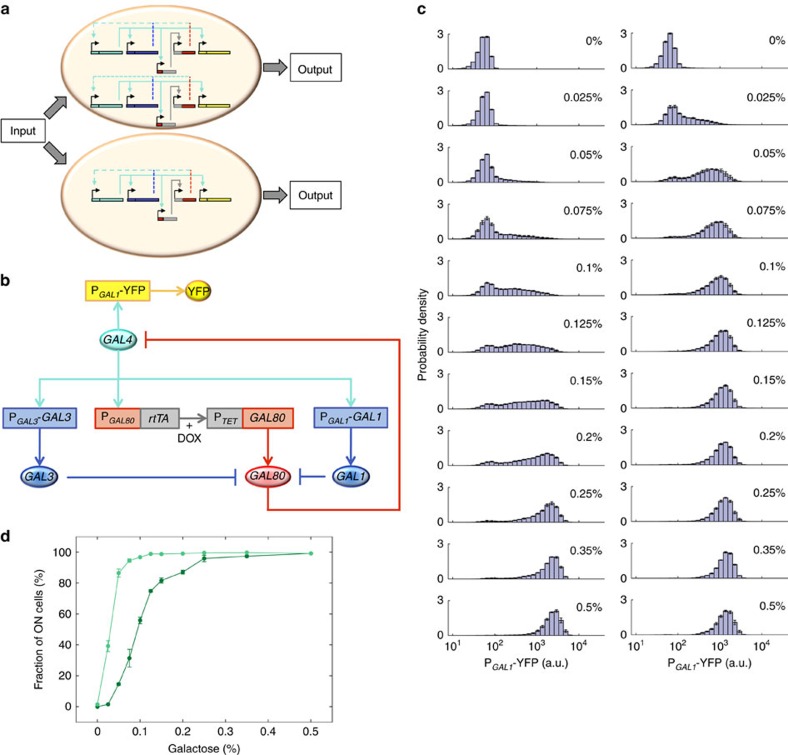
Topology and non-compensated response of the synthetic network. (**a**) Cells with one or two copies of the synthetic network. For each copy of the network, key network components (*GAL4, GAL3 & GAL1, GAL80*, P_*TET*_ & rtTA and P_*GAL1*_-YFP) are represented by turquoise, blue, red, grey and yellow, respectively. The regulation by *GAL4* is indicated by solid turquoise lines and feedback loops are shown by dashed lines. (**b**) Synthetic network architecture. Coloured ellipses depict proteins, while rectangles show the promoter-gene regions on DNA. Pointed turquoise/grey and blue/red arrows indicate activation and gene expression, respectively, while blunt arrows reflect inhibition. +DOX indicates the presence of doxycycline. (**c**) At different galactose concentrations, single-cell expression histograms show the response of diploid *S. cerevisae* strains carrying the synthetic GAL network topology. At left, diploid strain WP196 with two copies of the synthetic network; at right, diploid strain WP233 with one copy of the synthetic network. (**d**) Fraction of ON cells as a function of galactose concentration from diploid cells carrying one (WP233, light green) or two (WP196, dark green) copies of the synthetic network. Error bars indicate s.e.m. (*N*=4).

Comparing how noise in network output changes in networks with and without network-dosage compensation requires abolishing the compensated phenotype. One way to achieve this is by breaking the topology of the natural (compensated) GAL network. Therefore, we synthetically modified the network by integrating two copies of the P_*GAL80*_-*rtTA* and P_*TET*_-*GAL80* constructs into diploid yeast which was deleted in its endogenous *GAL80* genes (Fig. 2a,b). As previous work^25^ has shown that negative feedback regulation lowers noise in gene expression, we made sure that the *GAL80* negative feedback loop was still intact after our synthetic modification. We induced our synthetic strain with eleven different galactose concentrations and measured the P_*GAL1*_-YFP-reported activity of the GAL network in single cells (Fig. 2c, left column). In these experiments, we used a doxycycline concentration that was obtained by having the activity of the synthetic network match the activity of the wild-type diploid network in its linear-response regime (Supplementary Methods).

To find out how changing the dosage of this synthetic network affects the network activity, we halved the number of copies of the synthetic network in diploid cells, obtaining a yeast strain which was hemizygous in the synthetic network. This was done by deleting one copy of the *GAL2*, *GAL3*, *GAL1* and *GAL4* genes in addition to deleting both copies of the *GAL80* gene, followed by the integration of only one copy of the P_*GAL80*_-*rtTA* and P_*TET*_-*GAL80* constructs into diploid yeast genome. Next we induced our dosage-halved synthetic strain with several different galactose concentrations in the presence of the same doxycycline concentration as before (Supplementary Methods) for consistency, and measured the network activity in single cells. We analysed the resulting bistable expression distributions (Fig. 2c, right column) by quantifying the fraction of induced (ON) cells corresponding to network activity, and compared the numbers with the ones measured from the homozygous strain carrying two copies of the synthetic network (Fig. 2d). The activity of the synthetic network no longer exhibited network-dosage invariance (Supplementary Note 3), experimentally proving that the synthetic modifications we introduced to the network abolished its dosage-compensated behaviour.

### Mathematical analysis explains abolishment of compensation

To explain why insertion of a synthetic link causes the abolishment of compensation, we performed a mathematical analysis using a simple model (Supplementary Note 1) that considers the effect of global perturbations in the synthesis rates of the network components on the steady state activity level of the network. As shown in Supplementary Note 1A, the steady state activity of the natural GAL network is invariant to such perturbations. In contrast, we prove in Supplementary Note 1B that inserting the synthetic link as we did to such a network abolishes this invariance, as long as the level of expression from the TET promoter is dependent on the rtTA expression level—which is of course true for any inducible system. The activity of such a network, instead, is sensitive to global perturbations, and network-dosage changes are certainly large global perturbations.

### Noise level increases significantly in the synthetic network

**Figure 3:**
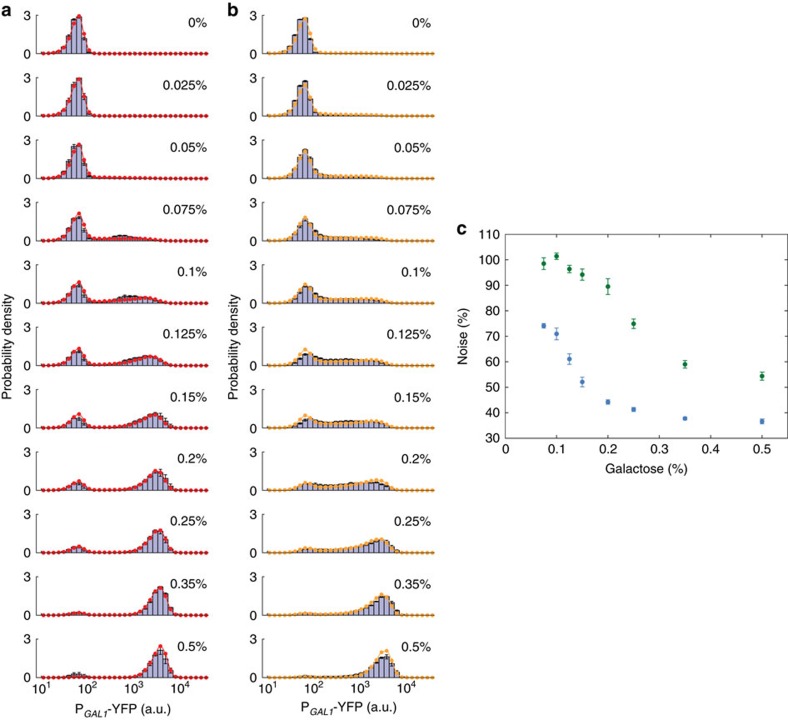
Noise reduction elucidated in haploid background and the model fitting. (**a**) Single-cell expression histograms showing the response of the natural (wild type) GAL network in haploid cells (MA0048, bars) at different galactose concentrations along with the fitted model output (red). (**b**) Single-cell expression histograms showing the response of the synthetic network in haploid cells (WP115, bars) at different galactose concentrations along with the fitted model output (orange). (**c**) Coefficient of variation for the expression level of ON cells in haploid cells carrying wild type (blue) or synthetic (green) networks at the given galactose concentrations. Error bars indicate s.e.m. (*N*=4).

To elucidate how noise in network output changes in networks with and without network-dosage compensation, we first used haploid strains carrying the natural or synthetic network topology. To obtain the strain carrying the synthetic network topology, we integrated one copy of P_*GAL80*_-*rtTA* and P_*TET*_-*GAL80* into haploid genome in *gal80Δ* background. We grew this strain and the haploid wild-type strain in several different galactose concentrations and then measured the P_*GAL1*_-YFP-reported activity of the synthetic and natural network by flow cytometry (Fig. 3a,b). For the synthetic strain, we added doxycycline into the growth media so that the activity of the synthetic network would match the activity of the wild-type haploid network in its linear-response regime (Supplementary Methods). Next, we quantified the noise in network activity by calculating the coefficient of variation of the single-cell gene expression distributions. For all noise analyses, we considered only the induced cells occupying the ON state of the bimodal profiles, as measurements from the OFF state cells primarily reflect cellular autofluorescence and the basal activity of the reporter, and would not shed any light on the noise in network activity, or lack thereof. Since the fractions of ON cells were naturally low at low-galactose induction levels (Fig. 3a,b), to obtain reliable noise values coming from the analysis of sufficient number of cells, we excluded from our analysis the expression data obtained from galactose concentrations <0.075%. Figure 3c shows the noise values obtained from our measurements and analyses. Wild-type haploid cells carrying the natural (dosage compensated) network indeed showed reduced noise levels compared with the noise obtained from the synthetic (non-compensated) network, verifying our hypothesis.

### The synthetic construct does not increase noise in isolation

Compared with the natural network, the synthetic network carries an additional link through the rtTA-P_*TET*_ system. Is the observed increase in noise due to having an additional link in the network or is it due to breaking network-dosage compensation? To rule out the former possibility, we inserted the same rtTA-P_*TET*_ synthetic regulatory link into a β-estradiol inducible system (Supplementary Fig. 5A,B) orthogonal to the GAL network (Supplementary Note 4). For this, we expressed in yeast a fusion protein^26^ composed of the oestrogen receptor (ER), an activation domain (AD) and the LexA DNA-binding domain. Using the P_*LexA*_-YFP reporter let us measure the monostable activity of the system in varying β-estradiol levels (Supplementary Fig. 5C,D). Then, to add the additional rtTA-P_*TET*_ synthetic regulatory link into this system, we constructed another yeast strain in which P_*LexA*_-rTTA and P_*TET*_-YFP constructs were integrated into the yeast genome in the presence of the LexA-ER-AD fusion protein. Using 0.35 μg ml^−1^ doxycycline gave rise to YFP expression levels similar to the ones observed from the system without the rtTA-P_*TET*_ link (Supplementary Fig. 5C,D). The presence of doxycycline and β-estradiol at the concentrations used did not substantially hamper the growth rates due to potential toxicity (Supplementary Fig. 5E). We next quantified noise in network activity using the two β-estradiol inducible strains with and without the additional rtTA-P_*TET*_ link. We saw that the presence of the additional link did not significantly increase the noise levels (Supplementary Fig. 5F), ruling out the possibility that it is the number of links in the GAL network that increases the noise.

### Stochastic simulation matches experimental observations

With the goal of having a quantitative understanding on our results, we developed a novel stochastic modelling approach and applied it to both natural and synthetic networks we constructed (Supplementary Methods). Since we were interested in elucidating the connection between network-dosage compensation and noise, it was essential to develop a reliable noise model at the gene network level. Differently from prior discrete stochastic modelling approaches^27^,^28^, our volume- and cell-cycle-aware model tracks the changes in cell volume, DNA replication and cell division, and dynamically adjusts rates of stochastic reactions based on this information. We first fitted our model to the experimental expression histograms obtained from haploid wild-type cells (Fig. 3a) by using a minimal set of free parameters corresponding to the transitions of the network promoters between the OFF and ON states and to the scale of action for network proteins; the rest of the parameters were fixed from literature (Supplementary Methods). The fit results were in good agreement with the single-cell fluorescence distributions across several galactose induction levels (Fig. 3a). Using the parameters obtained from this fit and the ones fixed from literature, we next fitted our model to the experimental expression histograms (Fig. 3b, Supplementary Fig. 7B) obtained from the haploid synthetic strain. In this fit, the only free parameters were the ones associated with the rtTA-P_*TET*_ system in addition to the scale of the YFP fluorescence (Supplementary Methods). Again, the fit results were in good agreement with the single-cell fluorescence distributions (Fig. 3b).

**Figure 4:**
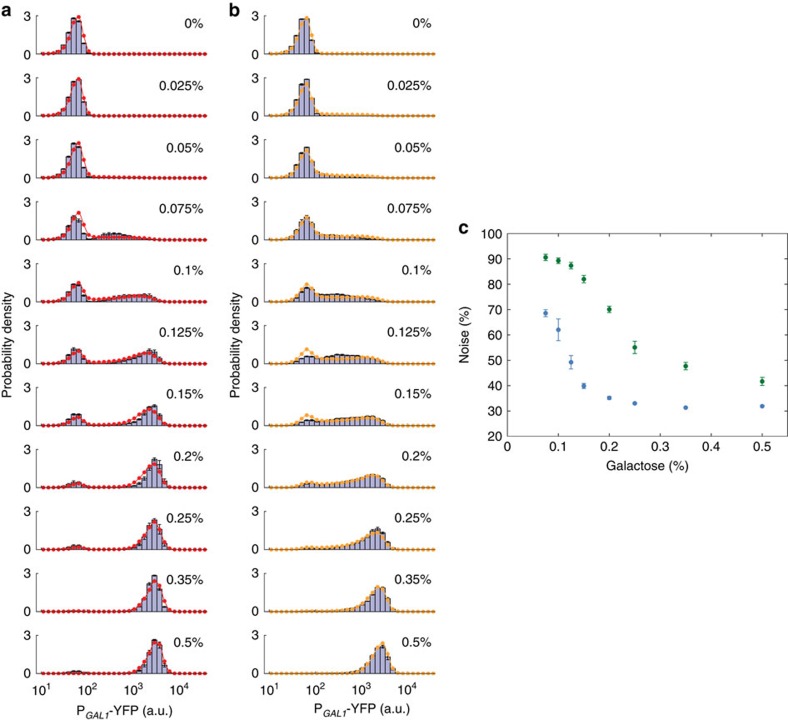
Noise reduction elucidated in diploid background and the model predictions. (**a**) Single-cell expression histograms showing the response of the natural (wild type) GAL network in diploid cells (WP128, bars) at different galactose concentrations along with the model prediction (red). (**b**) Single-cell expression histograms showing the response of the synthetic network in diploid cells (WP196, bars) at different galactose concentrations along with the model prediction (orange). (**c**) Coefficient of variation for the expression level of ON cells in diploid cells carrying wild type (blue) or synthetic (green) networks at the given galactose concentrations. Error bars indicate s.e.m. (*N*=4).

To test the predictive power of our model, we next simulated the activity of the natural and synthetic networks in diploid background for a time period matching the duration of our experimental inductions, 22 h (Supplementary Methods). In these simulations run separately for the natural and synthetic diploid networks, we did not use any free parameters; all parameters were fixed from literature or obtained from haploid fits (Fig. 3a,b). To implement the change from haploid to diploid background in our simulations, we doubled the cell volume as well as the number of copies of the regulatory genes in the network.In addition, for the synthetic diploid strain's simulation, we used two copies of P_*GAL80*_-*rtTA* and P_*TET*_-*GAL80* in the *gal80Δ* background. Figure 4a,b (red and orange circles) depicts the predicted network activity profiles at eleven different galactose induction levels, which are similar to the profiles obtained experimentally (Fig. 4a,b, histograms).

Using the stochastic model, we also simulated the behaviour of the natural and synthetic networks when their copy number is doubled in a haploid cell (Supplementary Note 5). The predictions are consistent with what we expected: network-dosage compensation is observed in the natural network, but not the synthetic (Supplementary Fig. 6).

### Noise reduction is also observed in diploid cells

Does the activity of the diploid natural (wild type) network also display noise-reduction compared with the activity of the diploid synthetic network? To address this question, we took the experimentally measured expression profiles obtained from the diploid homozygous strains carrying the natural and synthetic network topologies (Fig. 4a,b, histograms) and quantified noise in YFP-expressing cells as before. Similar to the results obtained from haploid cells, noise in network activity was indeed reduced for the natural network (Fig. 4c, blue circles) compared with the synthetic non-compensated network (Fig. 4c, green circles).

## Discussion

Even in a population of isogenic cells grown in the same environment, the expression level of a gene can vary significantly across cells due to noise in gene expression^2^,^6^,^7^,^8^,^9^. Depending on the cellular context, noise can be beneficial or detrimental to cellular activities. For example, noise was shown to confer an evolutionary advantage in fluctuating environments^13^, it was argued to be crucial to cellular development^29^, and was considered responsible for bacterial persistence through which population survival can be ensured^30^. On the other hand, noise can also be detrimental by introducing undesirable fluctuations in intracellular protein concentrations. Despite this, many key regulatory proteins in the budding yeast have been shown to have low-noise levels^31^, suggesting that cells implement noise reduction mechanisms when needed. What network design principles could cells use to reduce noise in network output? In this work, we hypothesized that dosage-compensating network topologies could act as noise-reduction modules in gene networks and verified our hypothesis by using natural and synthetic networks examined experimentally and computationally in haploid and diploid backgrounds. The frequently observed^5^ nature of the dosage compensated network topologies in natural gene networks suggests that cells take advantage of this noise-reduction capability in an ongoing basis, which would support the hypothesis that noise is a selectable trait tunable by evolution instead of being an inherent property of cellular machinery^32^,^33^.

## Methods

### Construction of plasmids and yeast strains

All *S. cerevisiae* strains used in this study have the W303 genetic background. Complete descriptions of all strains are in Supplementary Table 1. MA0048 is a haploid wild-type strain carrying the natural topology of the GAL network. This strain has the P_*GAL1*_-YFP reporter construct in its *ade2* locus. For this, *KpnI−P*_*GAL1*_*−BamHI* and *BamHI−YFP−EcoRI* fragments were cloned into pRS402 plasmid backbone upstream of the *CYC1* transcriptional terminator. The P_*GAL1*_ promoter sequence corresponds to the 669 base-pair region directly upstream of the start codon of the *S. cerevisiae GAL1* gene. The strains carrying the P_*GAL1*_-YFP reporter were built on MA0048 (Supplementary Table 1).

To construct the strains that are deleted in one or more network genes, specific markers (as described in Supplementary Table 1) were amplified (using PCR) from the plasmids carrying those markers by using 5′ and 3′ primers carrying 60 bp–80 bp homology to the region immediately up- and down-stream of the deleted gene. The transformed colonies were selected on plates missing the appropriate amino acid or carrying the appropriate antibiotics. The deletions were verified by PCR.

The haploid strain WP115 carrying the synthetic network topology was constructed by integrating one copy of the P_*GAL80*_-*rtTA* construct into the *ho* locus and one copy of P_*TET*_-*GAL80* into the *ura3* locus in a *gal80Δ* background. The *GAL80* promoter sequence used in this construct corresponds to the upstream intergenic region. In the synthetic constructs we used^12^, the synthetic *TET* promoter carried two binding sites for rtTA, the synthetic activator^16^.

To construct the diploid strain WP196 carrying two copies of the synthetic network, we mated the haploid synthetic strain WP115 with another haploid synthetic strain carrying one copy of the P_*GAL80*_-*rtTA* and P_*TET*_-*GAL80* construct into the *ho* and *ura3* loci, respectively, in a *gal80Δ* background.

To obtain the diploid strain WP233 carrying one copy of the synthetic network, we integrated one copy of the P_*GAL80*_-*rtTA* and P_*TET*_-*GAL80* construct into the *ho* and *ura3* loci, respectively (in a *gal80Δ* background) and deleted one copy of *GAL2, GAL3, GAL1, GAL4* genes by using the markers described in Supplementary Table 1.

The β-estradiol inducible strains (WP256 and WP258) were constructed in the following way. A plasmid carrying the P_*ACT1*_-[LexA-ER-B112] construct^26^ was obtained from Addgene (#58437) and integrated into the *his3* locus in both strains. To obtain WP258, we further integrated the P_*LexA*_-YFP construct into its *ura3* locus. The P_*LexA*_ promoter^26^ (Addgene #58435) carried eight LexA binding sites. To obtain WP256, we further integrated (i) P_*LexA*_-rTTA into the *ho* locus using KanMX as the marker and (ii) P_*TET*_-YFP into the *leu2* locus. The P_*TET*_ promoter carried two operator sites.

### Growth conditions and media

Cultures were grown in synthetic dropout media with the appropriate amino acid supplements. During the overnight growth period (22 h in 30 °C shaker), 2% raffinose was used as the carbon source. The overnight growth period was followed by the induction period (22 h in 30 °C shaker) with cultures containing 0–0.5% galactose in the presence of 0.08% glucose as non-inducing background sugar. After the induction period, the expression distributions of ∼10,000 cells were measured by flow cytometry (FACS-Verse; Becton Dickinson). Cell densities (OD_600_) at the end of the overnight and induction periods were ∼0.1. The culture volume was 5 ml during the overnight growth and induction periods.

### Cellular growth rate measurements

Cellular doubling times were measured after growing all strains (as described above) overnight followed by induction in different conditions. At the end of the 22 h induction period, frequent OD_600_ measurements were taken from exponentially growing cultures. Time dynamic growth curves were constructed and fitted to an exponential growth function to extract the doubling times of the strains in specific growth conditions (Supplementary Fig. 1).

### Analysis of experimental data

To minimize the effect of cell-size differences on our noise measurements, we gated the flow cytometry data using a small forward/side scattering gate. On average, ∼2,000 cells were included per experiment after the gating. Increasing the number of cells in the gate do not substantially affect the results (Supplementary Fig. 2). Fractions of ON cells were quantified using a cutoff determined from uninduced cell samples, and the mean and CV of the fluorescence of the ON cells were calculated using the same cutoff. For the analysis of the data obtained from the β-estradiol inducible strains, we gated the middle one-third of the forward and side scattering axes corresponding to each sample, leading to ∼4,700 gated cells on average. We obtained monostable YFP expression distributions and calculated noise (CV) of the YFP fluorescence. We observed that increasing the β-estradiol concentration from 0 to 300 nM increased the number of cells with relatively higher forward and side scattering values.

### Code availability

The code implementing the stochastic model is available from the corresponding author upon request.

### Data availability

The data that support the conclusions of this study are available from the corresponding author upon request.

## Additional information

**How to cite this article:** Peng, W. *et al*. Noise reduction facilitated by dosage compensation in gene networks. *Nat. Commun.* **7**, 12959 doi: 10.1038/ncomms12959 (2016).

## Supplementary Material

Supplementary Information Supplementary Figures 1-9, Supplementary Tables 1-6, Supplementary Notes 1-5, Supplementary Methods, Supplementary References
