# Supplementary material for: Noise reduction facilitated by dosage compensation in gene networks
Source: Nat Commun. 2016 Oct 3;7:12959. doi: 10.1038/ncomms12959 (PMC5063963; doi:10.1038/ncomms12959)
Supplement: Supplementary Information — Supplementary Figures 1-9, Supplementary Tables 1-6, Supplementary Notes 1-5, Supplementary Methods, Supplementary References [file ncomms12959-s1.pdf]

SUPPLEMENTARY TABLES

Supplementary Table 1. *Saccharomyces cerevisiae* strains used in the study. All strains carry the W303 genetic background.

Strain	Genotype
MA0048	*MATα*, *ade2*::*ADE2*-P$_{GAL1}$-*YFP*
WP115	*MATα*, *ade2*::*ADE2*-P$_{GAL1}$-*YFP, gal80Δ*::*KlTrpl*, *ho*::*HIS5*-P$_{GAL80}$-*rTTA*, *ura3*::*URA3*-P$_{TET}$-*GAL80*
WP128	*MATα/a*, *ade2*::*ADE2*-P$_{GAL1}$-*YFP/ade2*
WP232	*MATα/a*, *ade2*::*ADE2*-P$_{GAL1}$-*YFP/ade2*, *GAL2/gal2Δ*::*CaURA3*, *GAL3/ gal3Δ*::*HIS5*, *GAL4/gal4Δ*::*NatMX*, *GAL80/gal80Δ*::*KanMX, GAL1/gal1Δ*::*KlTrp1*
WP233	*MATα/a*, *ade2*::*ADE2*-P$_{GAL1}$-*YFP/ade2*, *leu2/leu2*::*LEU2*, *gal80Δ*::*KlTrpl/ gal80Δ*::*KlTrpl, GAL2/gal2Δ*::*CaURA3*, *GAL3/gal3Δ*::*HIS5*, *GAL1/gal1Δ*::*KlTrp1, GAL4/gal4Δ*::*NatMX*, *ho*::*HIS5*-P$_{GAL80}$-*rTTA/ho*, *ura3*::*URA3*-P$_{TET}$-*GAL80/ura3*
WP196	*MATα/a*, *ade2*::*ADE2*-P$_{GAL1}$-*YFP/ade2, leu2/leu2*::*LEU2*, *gal80Δ*::*KlTrpl/gal80Δ*::*KlTrpl*, *ho*::*HIS5*-P$_{GAL80}$-*rTTA/ho*::*HIS5*-P$_{GAL80}$-*rTTA*, *ura3*::*URA3*-P$_{TET}$-*GAL80/ura3*::*URA3*-P$_{TET}$-*GAL80*
WP256	*MATα, leu2::LEU2*-P$_{TET}$-*YFP, his3::HIS3*-P$_{ACT1}$-*[LexA-ER-B112], ho::KanMX-P$_{LexA}$-rTTA*
WP258	*MATα, ura3::URA3*-P$_{LexA}$-*YFP, his3::HIS3*-P$_{ACT1}$-*[LexA-ER-B112]*

Supplementary Table 2. Parameters for the volume module.

Parameter	Value	Unit
mean and SD of r_1	0.24 ± 0.06	fL min^{-1}
mean and SD of r_2	0.505 ± 0.108	fL min^{-1}
mean and SD of r_{2m}	0.007 ± 0.024	fL min^{-1}
mean and SD of *T1'*	11.7 ± 6.271	min
mean and SD of *T2*	14.4 ± 4.493	min
mean and SD of *T3*	50.175 ± 5.820	min
mean and SD of V_i	25 ± 5	fL
k	104.4	min
b	11.0	fL
c	0.25	

Supplementary Table 3. Fixed parameters for the GAL network in the gene network module.

Parameter	Value	Unit	References & Notes
$r'_{m,\ GAL3}$	0.7223	min^{-1}	[Note A]
$r'_{m,\ GAL80}$	0.1394	min^{-1}	[Note B]
$r'_{m,\ GAL1}$, $r'_{m,\ PGAL1\text{-}YFP}$	1.53828	min^{-1}	[Note C]
$r_{p,\ GAL3}$	20	min^{-1}	[Note A]
$r_{p,\ GAL80}$	4	min^{-1}	[Note B]
$r_{p,\ GAL1}$	20	min^{-1}	[Note D]
$r_{p,\ PGAL1\text{-}YFP}$	4	min^{-1}	[Note E]
$d_{m,\ GAL3}$	0.0330	min^{-1}	[1]
$d_{m,\ GAL80}$	0.02567	min^{-1}	[1]
$d_{m,\ GAL1}$	0.04077	min^{-1}	[1]
$d_{m,\ PGAL1\text{-}YFP}$	0.04077	min^{-1}	[Note F]
$d_{p,\ GAL3}$	0.03851	min^{-1}	[2]
$d_{p,\ GAL80}$	0.00024	min^{-1}	[2]
$d_{p,\ GAL1}$	0.00048135	min^{-1}	[Note G][3]
$d_{p,\ PGAL1\text{-}YFP}$	0.00048135	min^{-1}	[Note G]
b'_{GAL1}, $b'_{PGAL1\text{-}YFP}$	0.0018		[4]
b'_{GAL3}	0.07294		[4]
b'_{GAL80}	0.21739		[4]
S_{80}	30000		[Note H]
α	1		[5]
V_{ref}	50	fL	[Note I]

[Note A] Calculated using a basal level of 721 Gal3p/cell[6], basal transcription level[4] and protein half-life[2] from literature, and a translation rate of 20/mRNA/min.

[Note B] Calculated using a basal level of 784 Gal80p/cell[6], basal transcription level[4] and protein half-life[2] from literature, and a translation rate of 4/mRNA/min.

[Note C] Calculated from estimated mRNA abundance based on literature[4] and mRNA half-life measurement in galactose[1].

[Note D] Assigned based on estimated average translation rate[2,6,7] in yeast. Inaccuracies in this rate constant are accounted while fitting the scale parameter S_1.

[Note E] Arbitrarily assigned. Any inaccuracy is accounted for during the fluorescence fitting process.

[Note F] The degradation rate is assumed to be similar to that of *GAL1* mRNA. Any inaccuracy is accounted for during the fluorescence fitting process.

[Note G] This protein is highly stable and assigned a half-life of 24 hours.

[Note H] S_{80} is fixed at this arbitrarily assigned value only while fitting the GAL network parameters (see methods).

[Note I] Assigned based on the results from running the volume model.

Supplementary Table 4. Fixed parameters for the rtTA-TET network in the gene network module.

Parameter	Value	Unit	References & Notes
$r'_{\mathrm{m,\ TET\text{-}GAL80}}$	0.1	min^{-1}	[Note A]
$r_{\mathrm{p,\ rtTA}}$	4	min^{-1}	[Note A]
$d_{\mathrm{m,\ rtTA}}$	0.02567	min^{-1}	[Note A]
$d_{\mathrm{p,\ rtTA}}$	0.08	min^{-1}	[Note A]
γ	2		[Note B] [8]

[Note A] Arbitrarily assigned. Inaccuracies are accounted for during the fitting of the respective scales of action, S_d and S_{80}.

[Note B] γ is fixed at the value 2 as the active form of rtTA is a dimer.

Supplementary Table 5. Fitted parameters for the GAL network in the gene network module.

Parameter	Fitted value	Unit	Initial value	Lower bound	Upper bound
r_{GAL3}	0.0100	min^{-1}	0.01	0.01	10
r_{GAL80}	2.9995	min^{-1}	1	0.01	10
$r_{\mathrm{GAL1}}, r_{\mathrm{PGAL1\text{-}YFP}}$	0.1417	min^{-1}	0.1	0.01	10
f_{GAL3}	0.9899		0.9	0.01	0.99
f_{GAL80}	0.5048		0.05	0.01	0.99
$f_{\mathrm{GAL1}}, f_{\mathrm{PGAL1\text{-}YFP}}$	0.8811		0.9	0.01	0.99
S_3	38206.8		40000	20000	100000
S_1	6456.87		2000	100	6500
β	1.44155		2	1	4

Supplementary Table 6. Fitted parameters for the rtTA-TET network in the gene network module.

Parameter	Fitted value	Unit	Initial value	Lower bound	Upper bound
$r_{\mathrm{TET\text{-}GAL80}}$	0.0367	min^{-1}	0.05	0.01	5
$b'_{\mathrm{TET\text{-}GAL80}}$	0.0119		0.05	0.01	0.1
$f_{\mathrm{TET\text{-}GAL80}}$	0.5000		0.9	0.01	0.99
S_d	1.43864		0.75	0.001	2
S_{80}	109360		90000	10000	200000

SUPPLEMENTARY NOTE 1

Mathematical analysis on the activity of the GAL network and the synthetic network

The following mathematical analysis shows that the GAL network's activity is not sensitive to global perturbations such as network dosage changes or extrinsic noise, unlike the synthetic network. In other words, the GAL network has the capacity to buffer extrinsic noise, making the network activity display lower noise levels compared to the synthetic network.

A. Activity level of the GAL network is not sensitive to global perturbations

We use the following set of differential equations to describe the time evolution of the concentrations of proteins expressed from the GAL network genes:

$$\begin{cases} \dfrac{dx_1}{dt} = \theta_1\left[\lambda_1 + (1-\lambda_1)\big(f(x_1, x_3, x_{80}, g)\big)^{\eta_1}\right] - \gamma_1 x_1 \\ \dfrac{dx_3}{dt} = \theta_3\left[\lambda_3 + (1-\lambda_3)\big(f(x_1, x_3, x_{80}, g)\big)^{\eta_3}\right] - \gamma_3 x_3 \\ \dfrac{dx_{80}}{dt} = \theta_{80}\left[\lambda_{80} + (1-\lambda_{80})\big(f(x_1, x_3, x_{80}, g)\big)^{\eta_{80}}\right] - \gamma_{80} x_{80} \end{cases} \qquad (1)$$

In the above equations, x_n represents the average total concentration of the protein Gal*n*p, θ_n represents the maximal expression rate, λ_n represents the basal expression level, and η_n represents the nonlinearity of TF-promoter interaction for the gene GALn. γ_n represents the combined protein degradation/dilution rate constant of the protein Gal$n$p. $f(x_1, x_3, x_{80}, g)$ is the function representing the overall GAL network activity level and is defined as

$$f = \frac{1}{1 + \left(\dfrac{S_{80}x_{80}}{1 + (S_3 x_3 g + S_1 x_1 g)^{\alpha}}\right)^{\beta}} \qquad (2)$$

where α and β are nonlinearity coefficients for Gal1/3p-Gal80p interaction and Gal80p-Gal4p interaction, respectively, and S_i is the scale of action of the corresponding protein. Based on previous experimental observations[5], we know that Gal3p:Gal80p and Gal1p:Gal80p interacts with 1:1 stoichiometry, so we fixed α = 1. When $S_3 x_3 g + S_1 x_1 g \gg 1$, the form of f simplifies to

$$f = \frac{1}{1 + \left(\dfrac{S_{80}x_{80}}{S_3 x_3 g + S_1 x_1 g}\right)^{\beta}} \qquad (3)$$

This form has the important property

$$f(kx_1, kx_3, kx_{80}, g) = f(x_1, x_3, x_{80}, g) \text{ for all } k > 0 \tag{4}$$

We now introduce a perturbation factor $\delta > 0$ representing a global perturbation such as extrinsic noise or a network dosage change, so that the equations become

$$\begin{cases} \dfrac{dx_1}{dt} = \delta\theta_1\left[\lambda_1 + (1-\lambda_1)\big(f(x_1, x_3, x_{80}, g)\big)^{\eta_1}\right] - \gamma_1 x_1 \\ \dfrac{dx_3}{dt} = \delta\theta_3\left[\lambda_3 + (1-\lambda_3)\big(f(x_1, x_3, x_{80}, g)\big)^{\eta_3}\right] - \gamma_3 x_3 \\ \dfrac{dx_{80}}{dt} = \delta\theta_{80}\left[\lambda_{80} + (1-\lambda_{80})\big(f(x_1, x_3, x_{80}, g)\big)^{\eta_{80}}\right] - \gamma_{80} x_{80} \end{cases} \tag{5}$$

We now show that at steady state for equation set (5), the value of $f(x_1, x_3, x_{80}, g)$ is equal to the value of f at steady state for equation set (1).

Let the steady state values of x_1, x_3, and x_{80} for equation set (1) be X_1, X_3, and X_{80} respectively, therefore we have

$$\begin{cases} \theta_1\left[\lambda_1 + (1-\lambda_1)\big(f(X_1, X_3, X_{80}, g)\big)^{\eta_1}\right] - \gamma_1 X_1 = 0 \\ \theta_3\left[\lambda_3 + (1-\lambda_3)\big(f(X_1, X_3, X_{80}, g)\big)^{\eta_3}\right] - \gamma_3 X_3 = 0 \\ \theta_{80}\left[\lambda_{80} + (1-\lambda_{80})\big(f(X_1, X_3, X_{80}, g)\big)^{\eta_{80}}\right] - \gamma_{80} X_{80} = 0 \end{cases}$$

Multiplying both sides of each equation by δ yields

$$\begin{cases} \delta\theta_1[\lambda_1 + (1-\lambda_1)\big(f(X_1, X_3, X_{80}, g)\big)^{\eta_1} - \gamma_1 \delta X_1 = 0 \\ \delta\theta_3[\lambda_3 + (1-\lambda_3)\big(f(X_1, X_3, X_{80}, g)\big)^{\eta_3} - \gamma_3 \delta X_3 = 0 \\ \delta\theta_{80}[\lambda_{80} + (1-\lambda_{80})\big(f(X_1, X_3, X_{80}, g)\big)^{\eta_{80}} - \gamma_{80} \delta X_{80} = 0 \end{cases}$$

From (3) we know that $f(\delta X_1, \delta X_3, \delta X_{80}, g) = f(X_1, X_3, X_{80}, g)$, and therefore

$$\begin{cases} \delta\theta_1\left[\lambda_1 + (1-\lambda_1)\big(f(\delta X_1, \delta X_3, \delta X_{80}, g)\big)^{\eta_1}\right] - \gamma_1 \delta X_1 = 0 \\ \delta\theta_3\left[\lambda_3 + (1-\lambda_3)\big(f(\delta X_1, \delta X_3, \delta X_{80}, g)\big)^{\eta_3}\right] - \gamma_3 \delta X_3 = 0 \\ \delta\theta_{80}\left[\lambda_{80} + (1-\lambda_{80})\big(f(\delta X_1, \delta X_3, \delta X_{80}, g)\big)^{\eta_{80}}\right] - \gamma_{80} \delta X_{80} = 0 \end{cases} \quad (6)$$

Now consider that at steady state for equation set (3), we have

$$\begin{cases} \delta\theta_1\left[\lambda_1 + (1-\lambda_1)\big(f(x_1, x_3, x_{80}, g)\big)^{\eta_1}\right] - \gamma_1 x_1 = 0 \\ \delta\theta_3\left[\lambda_3 + (1-\lambda_3)\big(f(x_1, x_3, x_{80}, g)\big)^{\eta_3}\right] - \gamma_3 x_3 = 0 \\ \delta\theta_{80}\left[\lambda_{80} + (1-\lambda_{80})\big(f(x_1, x_3, x_{80}, g)\big)^{\eta_{80}}\right] - \gamma_{80} x_{80} = 0 \end{cases} \quad (7)$$

Comparing the two sets of equations (6) and (7), we can immediately see that $\delta X_1, \delta X_3, \delta X_{80}$ are the steady state values of x_1, x_3, and x_{80} for the system described by equation set (5), and that network activity level f is equal to $f(\delta X_1, \delta X_3, \delta X_{80}, g) = f(X_1, X_3, X_{80}, g)$, which is the network activity level of the system described by equation set (1) at steady state.

In other words, in this system, the network activity level f does not change in response to a global perturbation.

B. Activity of the synthetic network is sensitive to global perturbations

We describe the time evolution of the concentrations of proteins expressed from the synthetic network using a similar set of differential equations:

$$\begin{cases} \frac{dx_1}{dt} = \delta\theta_1\left[\lambda_1 + (1-\lambda_1)\big(f(x_1, x_3, x_{80}, g)\big)^{\eta_1}\right] - \gamma_1 x_1 \\ \frac{dx_3}{dt} = \delta\theta_3\left[\lambda_3 + (1-\lambda_3)\big(f(x_1, x_3, x_{80}, g)\big)^{\eta_3}\right] - \gamma_3 x_3 \\ \frac{dx_{\text{rtTA}}}{dt} = \delta\theta_{80}\left[\lambda_{80} + (1-\lambda_{80})\big(f(x_1, x_3, x_{80}, g)\big)^{\eta_{80}}\right] - \gamma_{\text{rtTA}} x_{\text{rtTA}} \\ \frac{dx_{80}}{dt} = \delta\theta_{\text{tet}}\left[\lambda_{\text{tet}} + (1-\lambda_{tet})\big(h(x_{rtTA}, d)\big)^{\eta_{\text{tet}}}\right] - \gamma_{80} x_{80} \end{cases} \quad (8)$$

In the above equations, θ_{tet}, λ_{tet}, η_{tet} are the maximum expression level, basal expression level, and TF-promoter interaction nonlinearity of the TET promoter, respectively. x_{rtTA} and γ_{rtTA} are the average total concentration and combined degradation/dilution rate constant of the rtTA protein. $h(x_{\text{rtTA}}, d)$ is a function representing the activity of the TET promoter as a function of rtTA level,

$$h = \frac{1}{1 + (S_{\text{rtTA}} d x_{\text{rttA}})^{-\varepsilon}} \qquad (9)$$

We claim that for any two distinct δ values $\delta_1 \neq \delta_2$, the value of f at steady state for the system described by equation set (8) are different, and we prove that by contradiction.

Suppose that the steady state values of x_1, x_3, x_{80} and x_{rtTA} for equation set (6) are X_1, X_3, X_{80} and X_{rtTA} when $\delta = \delta_1$, and Y_1, Y_3, Y_{80} and Y_{rtTA} when $\delta = \delta_2$. Since these are steady state values, we know that

$$\begin{cases} \delta_1\theta_1\left[\lambda_1 + (1-\lambda_1)\big(f(X_1, X_3, X_{80}, g)\big)^{\eta_1}\right] = \gamma_1 X_1 \\ \delta_2\theta_1\left[\lambda_1 + (1-\lambda_1)\big(f(Y_1, Y_3, Y_{80}, g)\big)^{\eta_1}\right] = \gamma_1 Y_1 \end{cases}$$

If $f(X_1, X_3, X_{80}, g) = f(Y_1, Y_3, Y_{80}, g)$, then dividing the two equations yields

$$\frac{\delta_1}{\delta_2} = \frac{X_1}{Y_1}$$

Let $k = \frac{\delta_2}{\delta_1} \neq 1$, then we have $Y_1 = kX_1$. Similarly, we can show that $Y_3 = kX_3$ and $Y_{\text{rtTA}} = kX_{\text{rtTA}}$. From the simplified form of f (3) and given the assumption $f(X_1, X_3, X_{80}, g) = f(Y_1, Y_3, Y_{80}, g)$,, we have

$$\frac{1}{1 + \left(\frac{S_{80}X_{80}}{S_3X_3g + S_1X_1g}\right)^{\beta}} = \frac{1}{1 + \left(\frac{S_{80}Y_{80}}{S_3kX_3g + S_1kX_1g}\right)^{\beta}}$$

It's easy to see that this equation holds only when $Y_{80} = kX_{80}$. Now consider the last equation in (6). We know that, since X and Y are steady state values,

$$\begin{cases} \delta_1\theta_{\text{tet}}\left[\lambda_{\text{tet}} + (1-\lambda_{\text{tet}})\big(h(X_{\text{rtTA}}, d)\big)^{\eta_{\text{tet}}}\right] = \gamma_{80} X_{80} \\ \delta_2\theta_{\text{tet}}\left[\lambda_{\text{tet}} + (1-\lambda_{\text{tet}})\big(h(Y_{\text{rtTA}}, d)\big)^{\eta_{\text{tet}}}\right] = \gamma_{80} Y_{80} \end{cases}$$

Substituting $Y_{80} = kX_{80}$, $Y_{\text{rtTA}} = kX_{\text{rtTA}}$, and $\delta_2 = k\delta_1$ yields

$$\begin{cases} \delta_1 \theta_{\text{tet}} \left[\lambda_{\text{tet}} + (1 - \lambda_{\text{tet}}) \left(h(X_{\text{rtTA}}, d) \right)^{\eta_{\text{tet}}} \right] = \gamma_{80} X_{80} \\ k\delta_1 \theta_{\text{tet}} \left[\lambda_{\text{tet}} + (1 - \lambda_{\text{tet}}) \left(h(kX_{\text{rtTA}}, d) \right)^{\eta_{\text{tet}}} \right] = \gamma_{80} kX_{80} \end{cases}$$

Which leads to (assuming $\lambda_{tet} \neq 1$ – i.e., the TET promoter is inducible)

$$h(X_{rtTA}, d) = h(kX_{rtTA}, d)$$

Since $k = \frac{\delta_2}{\delta_1} \neq 1$, this requires h, or the activity of the TET promoter, to be independent of the level of rtTA, which cannot be true for any sensible form of h, and is certainly false for the form we used.

Hence, the assumption $f(X_1, X_3, X_{80}, g) = f(Y_1, Y_3, Y_{80}, g)$ must be incorrect, and for any two distinct δ values $\delta_1 \neq \delta_2$, the value of f at steady state for the system described by equation set (8) must be different. Thus, unlike the wild-type GAL network, the activity of the synthetic network is sensitive to global perturbations.

SUPPLEMENTARY NOTE 2

Validation of experimental parameters

A. The synthetic construct is not toxic under the conditions used

As there have been reports of rtTA-induced toxicity effects[9,10], we measured the doubling times of all strains by using 0.5% galactose, the highest concentration used in this study, which would be expected to produce the highest levels of rtTA in the synthetic strains. For the synthetic strains, doubling times with and without doxycycline were measured. All strains/conditions displayed the expected doubling time (~90min) and we observed no toxicity effects under the conditions used (Supplementary Fig. 1).

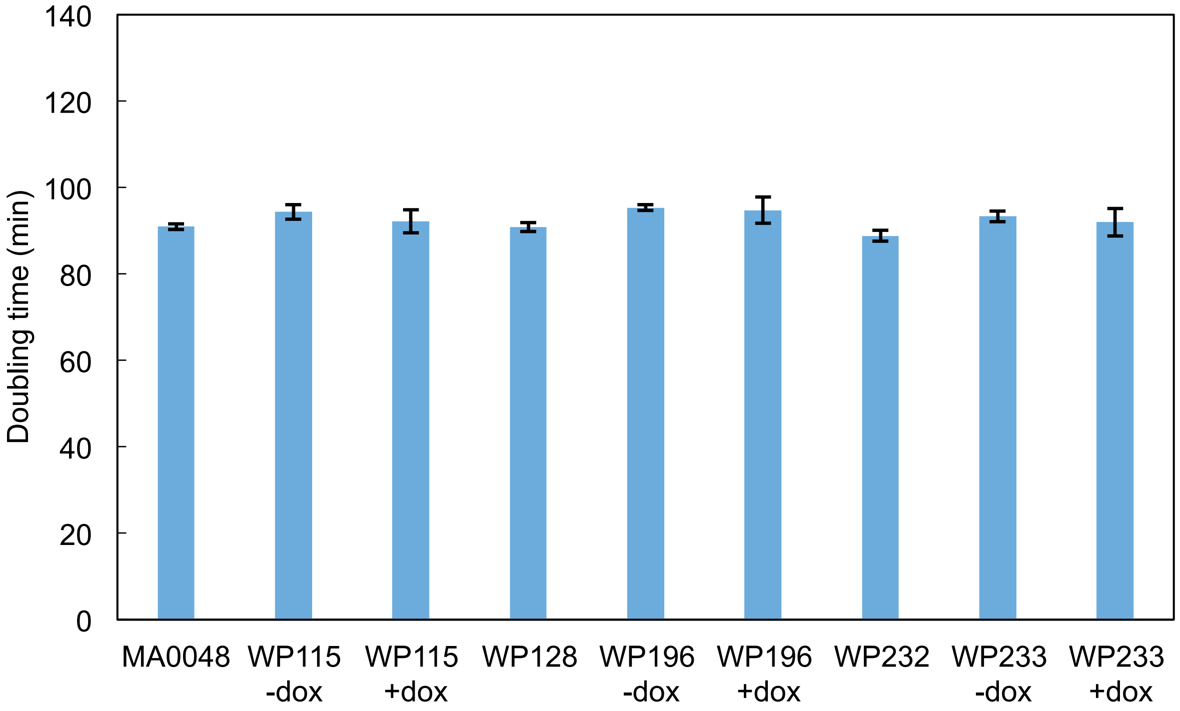

Supplementary Figure 1. Doubling time measurements for all strains. After 22 hours of induction using 0.5% galactose, doubling times were measured by taking frequent OD measurements from the continuously growing cultures. For the synthetic strains (WP115, WP196, WP233), doubling times both with (+dox) and without (-dox) doxycycline were measured by using the appropriate doxycycline concentrations (Supplementary Fig. 7) in addition to 0.5% galactose. Error bars indicate s.e.m. (N=2).

B. Increasing the sample size for flow cytometry did not substantially affect the expression distributions

To test the effects of increasing the sample size, we induced strains carrying the wild-type (WP128) and synthetic (WP196) topology at 0.15% galactose for 22 hours, increased the sample size tenfold so as to result in about ~20,000 cells on average after gating, and compared the distributions obtained with those obtained using the sample size of ~2,000 cells. No substantial difference was observed between the two distributions (Supplementary Fig. 2).

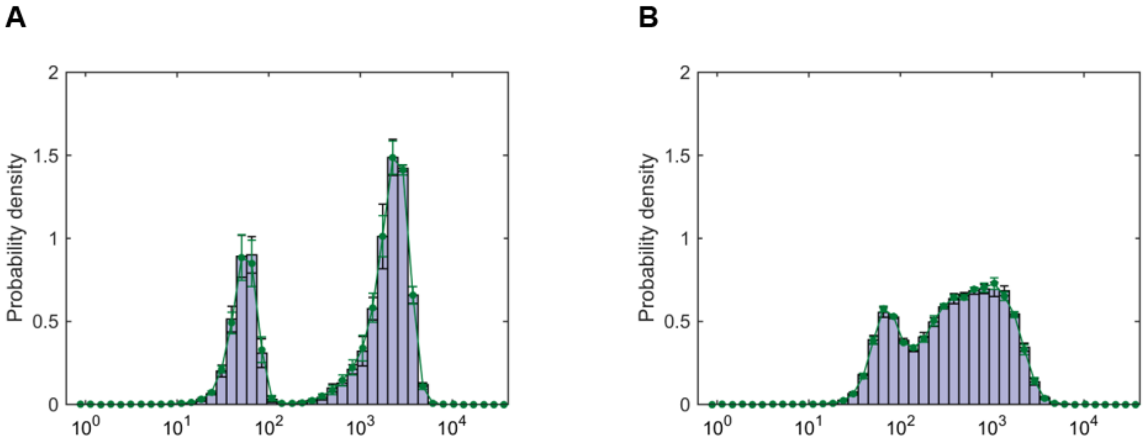

Supplementary Figure 2. Increasing the sample size has no significant effect on expression distributions. WP128 (**A**) and WP196 (**B**) strains were induced in 0.15% galactose and two samples were taken, one resulting in ~2,000 cells after gating (bars) and one resulting in ~20,000 cells after gating (points). No significant difference was observed between the two distributions. Error bars indicate s.e.m. (N ≥ 2).

C. Stability of the expression levels at 22 hours of induction

To show that 22 hours of induction is sufficient for the gene expression profiles to stabilize, we induced strains carrying the wild-type (WP128) and synthetic (WP196) topology with a linear-region galactose concentration (0.15% galactose) for either 22 hours or 27 hours. The resulting expression distributions were similar at the two time points for both strains (Supplementary Fig. 7).

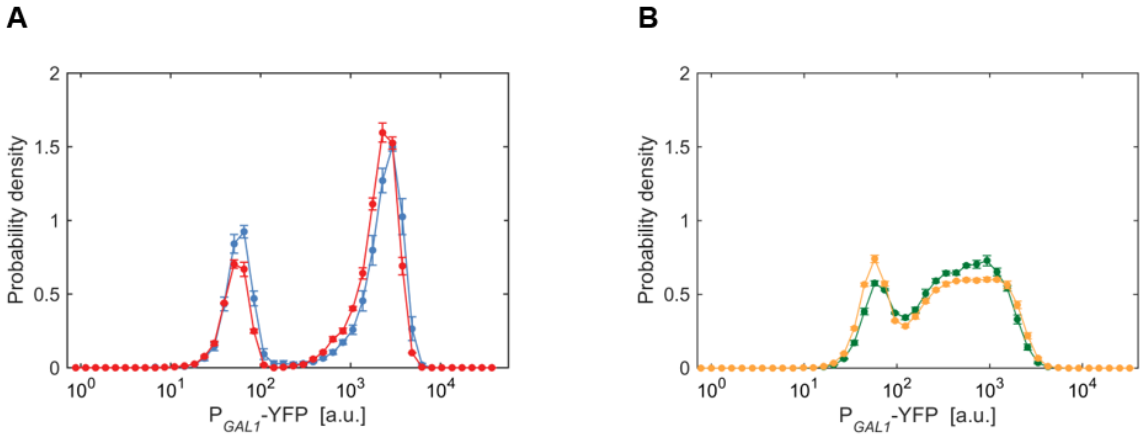

Supplementary Figure 3. Stability of the expression levels. Strains carrying the wild-type (WP128) (**A**) and synthetic (WP196) (**B**) network were induced with 0.15% galactose. Samples were taken at two time-points: at 22 hours after induction (blue/green) and at 27 hours after induction (red/orange), and expression profiles were measured with FACS. Error bars indicate s.e.m. (N ≥ 2).

SUPPLEMENTARY NOTE 3

Quantification of the level of network dosage compensation.

We quantified the level of dosage compensation by averaging the absolute difference in induction level between strains with one and two copies of the gene network across all inducer levels (Fig. 1D, Fig. 2D). Under this metric, lower scores indicate stronger dosage compensation effects. As seen in Supplementary Fig. 4, the compensation score for the wild-type network (Fig. 1D) is only 5%, as compared to the 25% for the synthetic network (Fig. 2D). When we excluded the basal (0% galactose) and the saturation (0.35% and 0.5% galactose) regions, the score for the synthetic network was even higher at ~35% (Supplementary Fig. 4B).

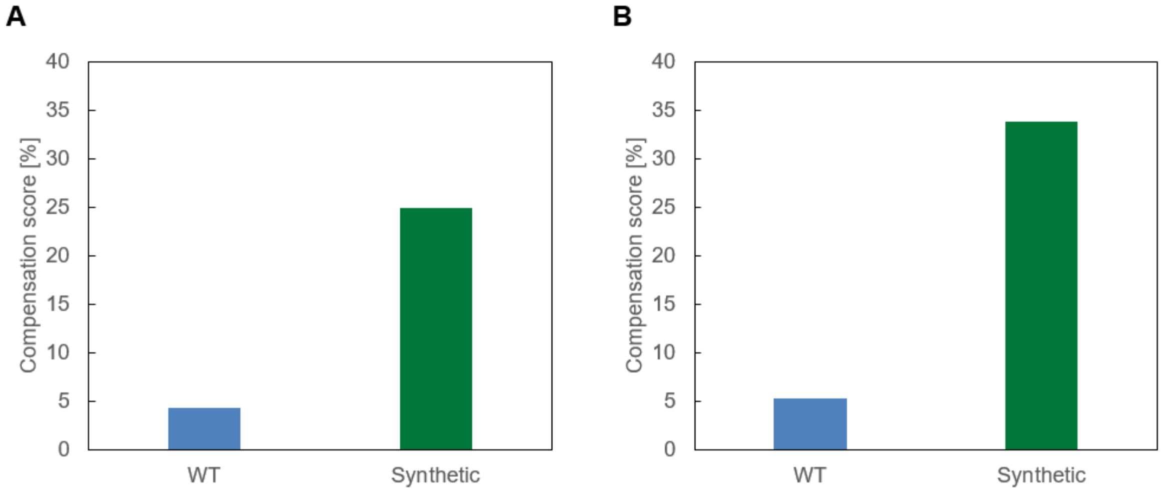

Supplementary Figure 4. Compensation score of the WT and synthetic network. The score is calculated by taking the mean of the absolute difference between the fraction of ON cells in strains carrying one or two copies of the network across all galactose concentrations (**A**) or all concentrations except 0%, 0.35%, 0.5% (**B**). 'WT' corresponds to WP128/WP232 (Fig. 1D), while 'synthetic' corresponds to WP196/WP233 (Fig. 2D). Lower score indicates higher level of network dosage compensation.

SUPPLEMENTARY NOTE 4

The β-estradiol inducible genetic system

The strains carrying the β-estradiol inducible system (WP256 and WP258) were grown in synthetic dropout media with the appropriate amino-acid supplements. During the overnight growth period (22 hours in 30°C shaker), 2% glucose was used as the carbon source. The overnight growth period was followed by the induction period (22 hours in 30°C shaker) with cultures containing 0-300nM β-estradiol in the presence of 2% glucose. Cultures carrying the strain WP256 also contained 0.35μg/mL doxycycline. After the induction period, the expression distributions of approximately 4,700 gated cells were measured by flow cytometry (FACS-Verse; Becton Dickinson), obtaining monostable YFP expression distributions. Cell densities (OD_{600}) at the end of the overnight and induction periods were between 0.3-0.7. The culture volume was 5ml during the overnight growth and induction periods.

Using a haploid strain without YFP, we measured cellular autofluorescence, subtracted it from all expression levels, and plotted the results (Supplementary Fig. 5C). We observed 8-9 fold induction in both WP256 and WP258 (Supplementary Fig. 5D). For stringency of the comparison, it is important to make sure that the fold-induction range of the estradiol system used here is equal to or more than the fold-induction range of the P_{GAL80} promoter. Previous work[4] has shown that the P_{GAL80} promoter is inducible ~5-fold at full induction, validating our choice of the estradiol and doxycycline concentrations for a reliable noise comparison at ranges comparable to the range of rtTA-TET induction under the control of the P_{GAL80} promoter.

To find out the growth rates of the two strains in the absence and presence of β-estradiol, we measured cellular doubling times after growing the two strains (as described above) overnight followed by induction in different conditions (Supplementary Fig. 5E). At the end of the 22 hours induction period, frequent OD_{600} measurements were taken from exponentially growing cultures. Time dynamic growth curves were constructed and fitted to an exponential growth function, leading to the extraction of the doubling times of the strains in specific growth conditions (Supplementary Fig. 5E). In the absence of estradiol, the two strains grew at similar rates (with ~105 min doubling time due to fusion-protein expression). In the presence of 300nM β-estradiol, the doubling times were approximately 130min. Despite the slight increase in doubling times, both WP256 and WP258 grew at similar rates, making the noise comparisons between the two strains reliable.

For both strains, noise levels were quantified (Supplementary Fig. 5F) across different β-estradiol concentrations by calculating coefficient of variation values obtained from the experimental YFP expression distributions measured by flow cytometry. We saw that the

presence of the additional rtTA-TET link did not significantly increase the noise levels (especially in the induction range of the P_{GAL80} promoter), ruling out the possibility that it is the number of links in the GAL network that increases the noise.

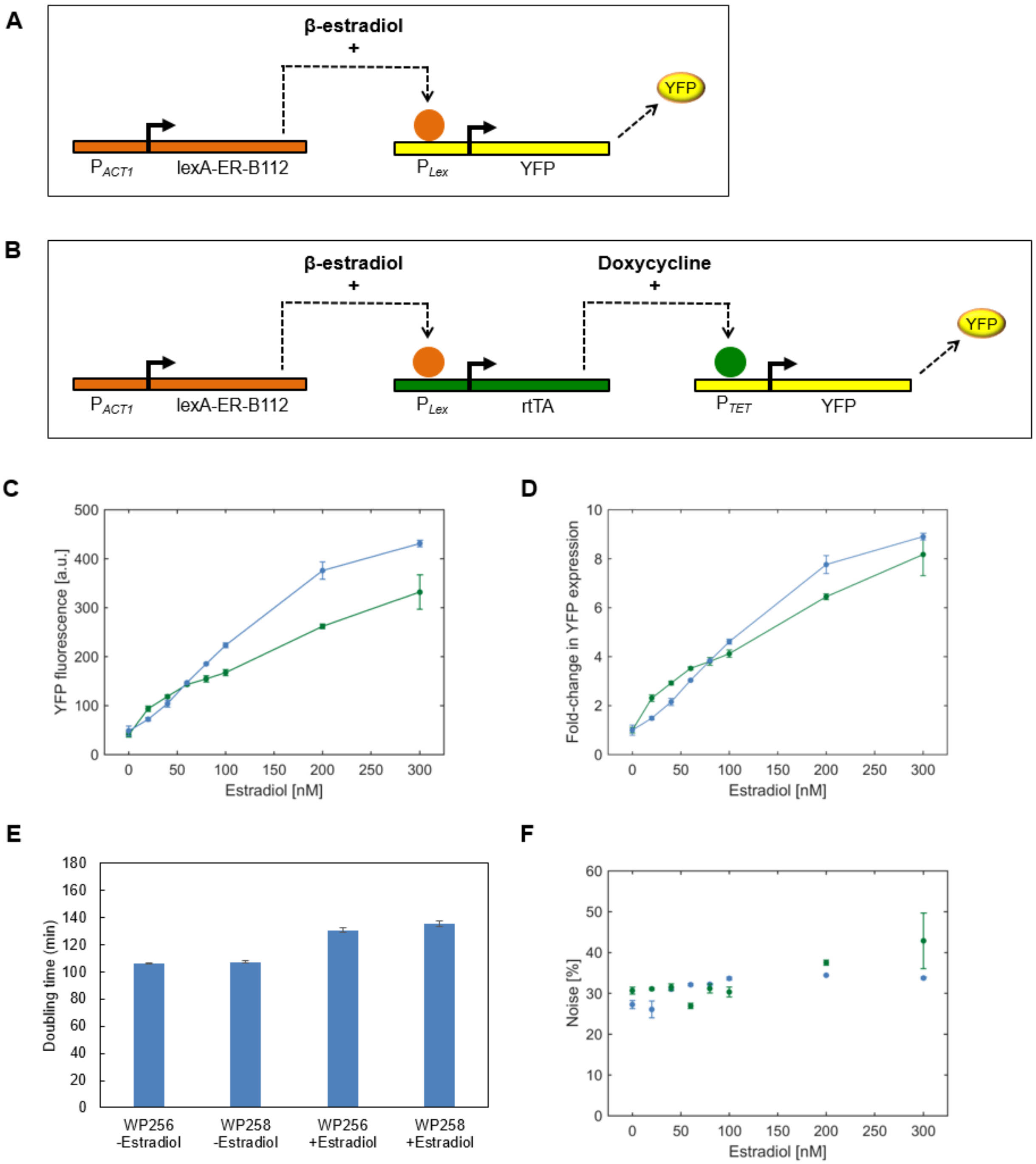

Supplementary Figure 5. The β-estradiol inducible genetic system. (**A-B**) The genetic architecture of the strain WP258 (A) and WP256 (B). (**C**) YFP expression levels across different β-estradiol concentrations. Blue, WP258; green, WP256. Error bars are s.e.m. (N=3). (**D**) Relative to the YFP expression level obtained at 0nM β-estradiol for each strain (C), fold-changes in YFP expression were quantified for each strain and plotted across the β-estradiol concentrations (blue, WP258; green, WP256). Error bars are s.e.m. (N=3). (**E**) Doubling times of the strains WP256 and WP258 in the absence and presence of 300nM β-estradiol. Cultures with WP256 also contained 0.35μg/mL doxycycline. Error bars

are s.e.m. (N=2). (**F**) Noise levels across different β-estradiol concentrations (blue, WP258; green, WP256). For noise quantification, coefficient of variation (CV) values were calculated using the experimental YFP expression distributions obtained by flow cytometry. Error bars are s.e.m. (N=3).

SUPPLEMENTARY NOTE 5

Simulating the network activity levels when network copy number is doubled in haploid cells

To see how adding a second copy of the wild type or synthetic network affects the network activity levels and compensation in haploid cells, we performed simulations using the parameter values listed in Supplementary Tables 2-6 and compared the results to the ones obtained experimentally from haploid strains carrying one copy of the wild type (MA0048) or synthetic (WP115) network (Supplementary Fig. 6). Compensation scores were calculated as described in Section VIII with a lower score indicating a higher level of network dosage compensation. As expected, the synthetic network displayed a low level of network dosage compensation.

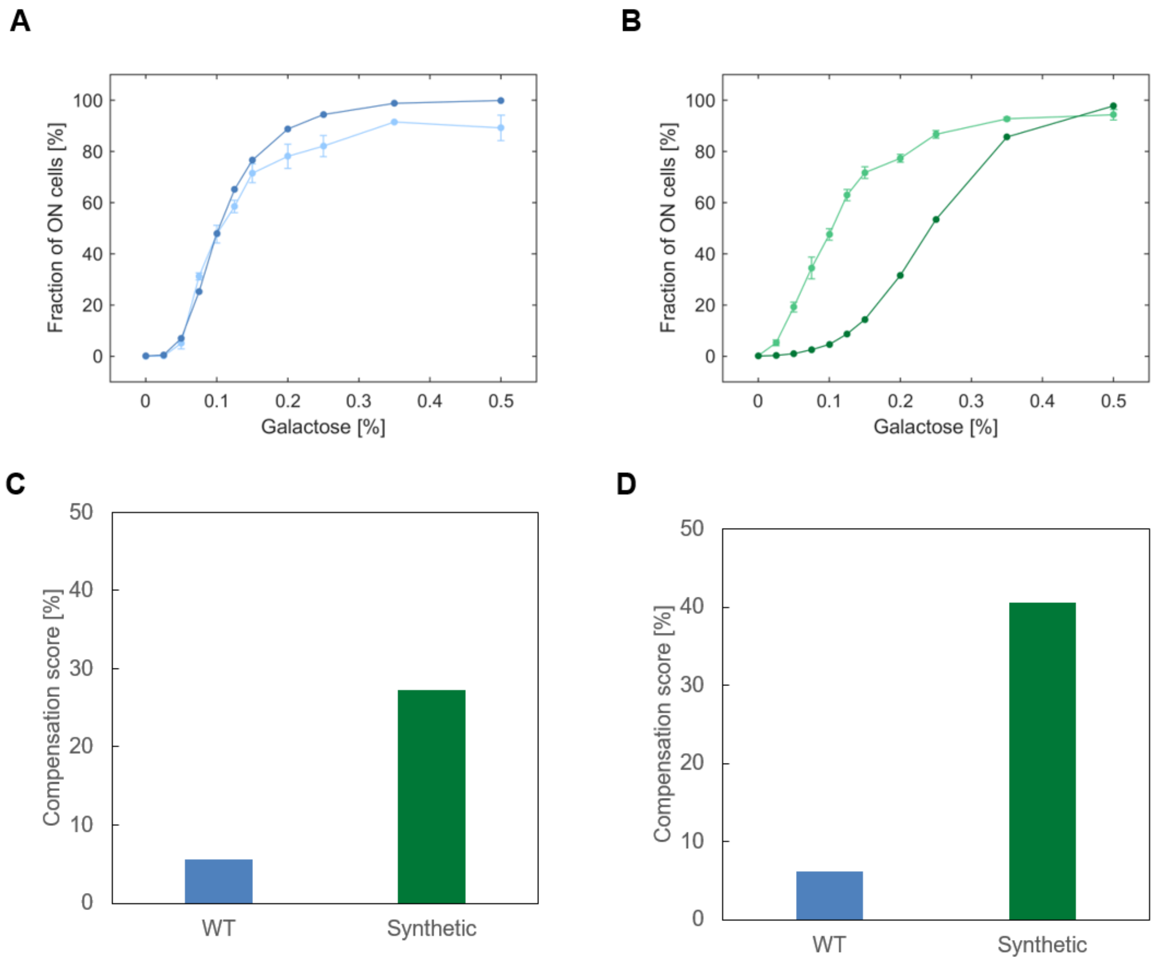

Supplementary Figure 6. Simulating the network activity levels when network copy number is doubled in haploid cells. (**A**) Experimental fraction of ON cells as a function of galactose concentration from haploid cells carrying one copy of the GAL network (MA0048, light blue; error bars indicate s.e.m. (N=4)), compared to simulated haploid cells containing two copies of the wild-type GAL network (dark blue). (**B**) Experimental fraction of ON cells as a function of galactose concentration from haploid cells carrying one copy of the synthetic network (WP115, light green; error bars indicate s.e.m. (N=4)), compared to simulated haploid cells containing two copies of the synthetic network (dark green). The simulations in (A-B) were performed using the parameter values listed in Tables S2-S6. **(C-D)** Compensation scores for WT and synthetic networks, calculated using the data in (A-B), across all galactose concentrations (**C**) or all concentrations except 0%, 0.35%, and 0.5% (**D**). Compensation scores are calculated as described in Section VIII. 'WT' corresponds to MA0048 and its simulation with two copies of the wild type network (A), while 'synthetic' corresponds to WP115 and its simulation with two copies of the synthetic network (B). A lower score indicates a higher level of network dosage compensation.

SUPPLEMENTARY METHODS

I. Determining the doxycycline concentrations used for the synthetic strains

To determine the doxycycline concentration that would cause the activity of synthetic network to match that of the wild-type GAL network, we used strains WP196 and WP115, and measured the P_{GAL1}-YFP expression levels in cells induced with 0.1% galactose and various doxycycline concentrations. The doxycycline concentrations (0.231μg/mL for WP196 and 0.296μg/mL for WP115) used in the subsequent experiments were determined by linear interpolation using nearby data points to match the fraction of ON cells produced by the corresponding wild-type strain (WP128 for WP196, MA0048 for WP115) (Supplementary Fig. 7).

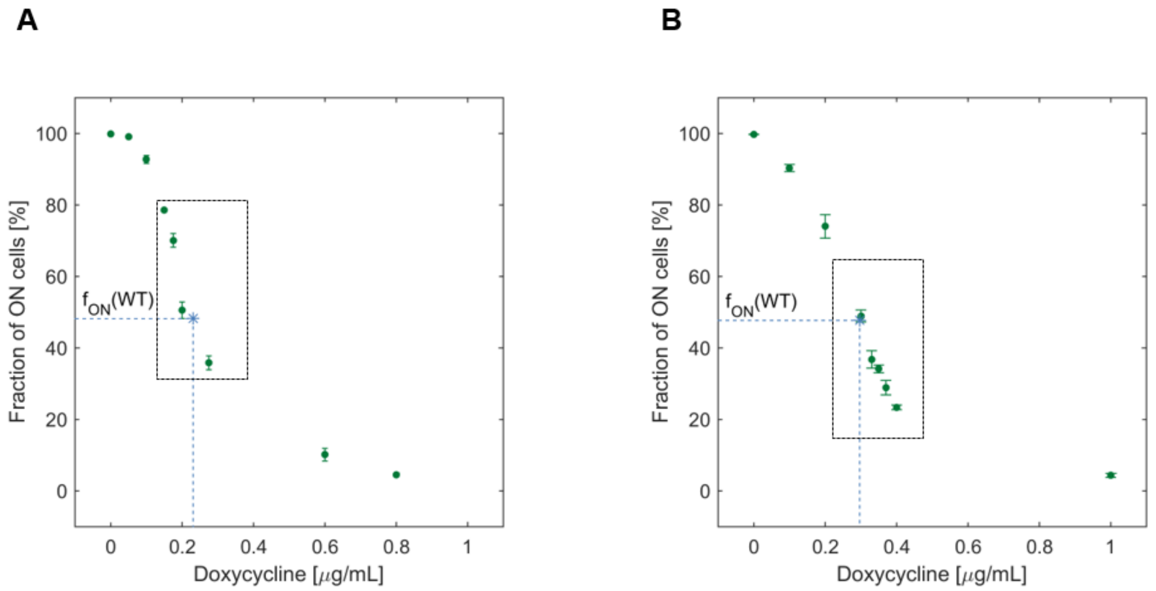

Supplementary Figure 7. Interpolation to determine the doxycycline concentrations used. Diploid (**A**, WP196) and haploid (**B**, WP115) strains carrying the synthetic network were induced with 0.1% galactose and various doxycycline concentrations and the fraction of ON cells were quantified (green dots). The doxycycline concentrations used in subsequent experiments (blue asterisk; **A,** 0.231 μg/mL; **B**, 0.296 μg/mL) were determined by linear interpolation using nearby data points (indicated in dashed box) to match the fraction of ON cells produced by the corresponding wild type strain (dashed blue line; **A**, WP128; **B**, MA0048). Error bars indicate s.e.m. (N=3).

II. Building a volume- and cell-cycle-aware stochastic model to predict noise in network activity

The volume- and cell-cycle-aware stochastic model we built contains two interacting modules. The first module is for cell volume growth and division, while the second module is for a multi-component gene network.

A. The volume module

The volume module models cell volume growth and division during cell cycle. Based on previous experimental characterization[11] on the budding yeast cell cycle, the cell grows linearly in the G1 and S/G2/M stages but at different rates. The G1 phase is further divided into two time blocks (T1 and T2), corresponding respectively to the time from the beginning to G1 to *start* and the time from *start* to end of G1 (Supplementary Fig. 8). The previous work has shown that the volume at *start* is linearly related to the G1 phase growth rate such that the duration of T1 obeys the following equations:

$$T1 = \min\left(T1', \frac{V_{\mathrm{s}} - V_0}{r_1}\right), \qquad V_s = k\, r_1 + b$$

where $T1'$ is a lower bound to the length of $T1$, V_0 is the volume of the cell at the beginning of the cell cycle, V_s is the volume of the cell at *start*, r_1 is the rate of volume growth in G1, and k and b are model parameters relating r_1 to V_{s}. The experimentally characterized model parameters are the mean and standard deviations of the initial volume of the starting cells (V_{i}), the growth rate in G1 (r_1), the overall growth rate in S/G2/M (r_2), the mother compartment's growth rate in S/G2/M ($r_{2\mathrm{m}}$), the minimum length of T1 ($T1'$), the duration of T2 ($T2$), and the duration of S/G2/M ($T3$), each of which was assumed to follow a normal distribution, along with k and b.

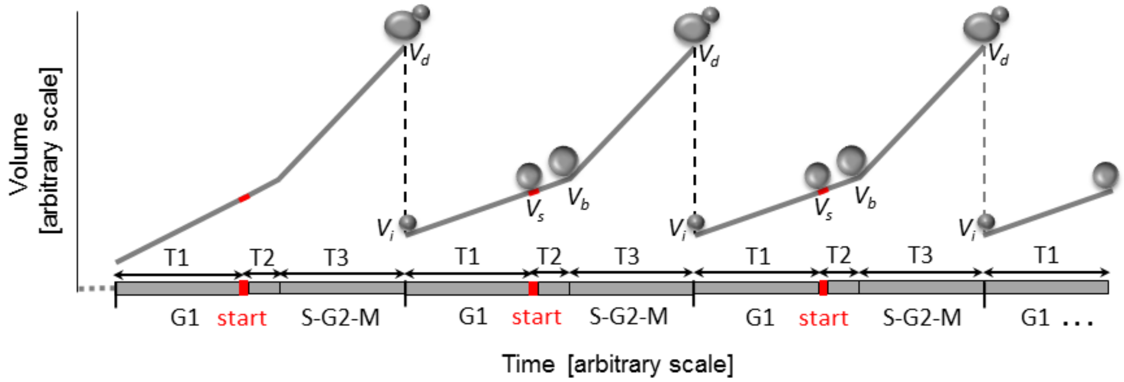

Supplementary Figure 8. Illustration of the volume model. The cell cycle is divided into two stages: G1 and S-G2-M, with the cell growing at a different rate in each stage. G1 is further divided into T1 and T2, separated by *start*. The volume increase in S-G2-M is mostly attributable to the growth of the daughter compartment.

At each cell division, a daughter cell was assumed to partially inherit its parent's parameter values. The exact level of inheritance is described by an additional model parameter, c, such that for a given parameter p,

$$p_{\mathrm{daughter}} = c\, p_{\mathrm{parent}} + (1 - c)\, p_{\mathrm{fresh}}$$

where p_{fresh} is a value sampled from the distribution of p.

B. The gene network module

We define a gene network as a group of genes whose promoters are all under the control of the same master transcription factor. Thus, in wild-type cells, *GAL80, GAL3,* and *GAL1* are all in the same gene network, while in the cells carrying the synthetic construct, P_{GAL80}-rtTA, *GAL3,* and *GAL1* belong to one gene network, while P_{TET}-*GAL80* is in its own network.

The activity of each network is described using a functional form that relates the concentration of activators, inhibitors, and inducer to the current activity level. The activity of the rtTA-TET network in synthetic strains is described using a Hill function[12]:

$$F_{\mathrm{TET}} = \frac{1}{1 + (S_{\mathrm{d}}\, d\, [rtTA])^{-\gamma}}$$

where S_{d} is a scaling factor, d is the inducer (doxycycline) concentration, and γ a nonlinearity coefficient.

The GAL network activity is described using the following functional form, which is derived (see section I, below) from the interactions among the network components:

$$F_{GAL} = \frac{1}{1 + \left(\frac{S_{80}\,[\text{Gal80p}]}{1 + (S_3\, g\, [\text{Gal3p}] + S_1\, g\, [\text{Gal1p}])^{\alpha}} \right)^{\beta}}$$

where S_1, S_3, and S_{80} are scaling factors, g is the inducer (galactose) concentration, and α and β nonlinearity coefficients.

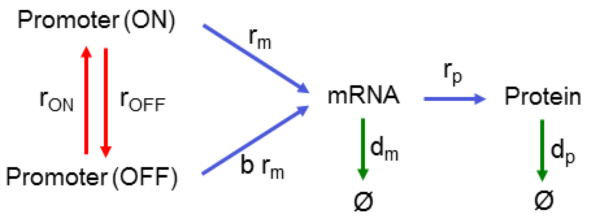

Supplementary Figure 9. The seven stochastic reactions for each gene. The promoter switches between OFF and ON states. mRNA is produced at the max rate from the ON state only, but the off state also produces some mRNA. mRNA is then translated into protein, and both mRNA and protein can be degraded.

For each gene in the network, we construct a set of seven stochastic reactions as illustrated in Supplementary Fig. 9. Each gene's promoter is assumed to switch between two states, OFF and ON. Using R to denote the number of mRNA copies, P the number of protein copies, PR_{OFF} (PR_{ON}) the number of OFF (ON) promoter copies, V the cell volume, we describe the reactions as:

Promoter activation………………………. $r_{\text{ON}}\, PR_{\text{OFF}}$

Promoter inactivation…………………..….$r_{\text{OFF}}\, PR_{\text{ON}}$

mRNA synthesis from OFF promoter……$r_{\text{m}}\, b\, PR_{\text{OFF}}\, \frac{V_{\text{ref}}}{V}$

mRNA synthesis from ON promoter……..$r_{\text{m}}\, PR_{\text{ON}}\, \frac{V_{\text{ref}}}{V}$

mRNA degradation…………………….....$d_{\text{m}}\, R\, \frac{V_{\text{ref}}}{V}$

Protein synthesis………………………….$r_{\text{p}}\, R\, \frac{V_{\text{ref}}}{V}$

Protein degradation…..……………………$d_{\text{p}}\, P\, \frac{V_{\text{ref}}}{V}$

where V_{ref} is a constant scaling factor equal to the average cell volume in the population, introduced so that the values of the rate parameters more closely match experimental measurements. These seven stochastic reactions are governed by the parameters r_{OFF}, r_{ON}, r_{m}, b, r_{p}, d_{m}, and d_{p}. The rate parameter r_{ON} is determined by the following equation:

$$r_{\mathrm{ON}} = r\, F^{\eta}$$

where r is a model parameter representing the maximum activation rate of the promoter of the gene at issue, F is the current value of the functional form of the gene network containing the gene at issue, and η a nonlinearity coefficient characterizing the interaction between the gene network's master transcription factor and the gene's promoter.

We chose to parameterize promoter transitions using the fraction of time a promoter spends in the active state when fully induced, denoted by f, rather than the promoter inactivation rate r_{OFF}. We define f as

$$f = \frac{r}{r + r_{\mathrm{OFF}}}$$

Further, to facilitate using parameter values obtained from existing experimental measurements, we use the observed mRNA synthesis rate r'_{m} instead of the actual mRNA synthesis rate r_{m}, the difference being that the observed rate reflects the fact that even in fully induced cells, a gene is only being actively transcribed a fraction (f) of the time. The two are related by the equation

$$r'_{\mathrm{m}} = r_{\mathrm{m}}\, f + b\, r_{\mathrm{m}}\, (1 - f)$$

Similarly, we use the observed basal expression level b', instead of the actual ratio between OFF-state and ON-state transcription rates b. The two are related by the equation $b'\, r'_{\mathrm{m}} = b\, r_{\mathrm{m}}$. To summarize, for each gene (including the reporter), there are seven stochastic reactions described by eight parameters: r, η, f, r'_{m}, b', r_{p}, d_{m}, and d_{p}.

To simulate global transcriptional noise, for each individual cell a random perturbation is applied to each rate parameter. Rate parameters for the same process (e.g., translation) are perturbed by the same fraction for all genes in the cell.

C. Interaction between the two modules

Cell volume growth is implemented by adding an additional stochastic reaction into the set of stochastic reactions specified by the gene network module; this reaction fires at a rate determined by the cell volume module, and each time the reaction fires, the cell volume is

incremented by a small amount. The change in cell volume, in return, will cause the rates of all the other stochastic reactions to change.

To model DNA replication during the cell cycle, upon entry into the S/G2/M phase we double the count of all promoters, on the assumption that an ON promoter would replicate into two ON ones, while an OFF promoter would replicate into two OFF ones.

For cell division, all mRNA and protein contents are distributed between mother and daughter cells following a binomial distribution based on the volume ratio between the mother and daughter compartments.

D. *Determining model parameter values from literature*

For the volume module, all model parameters except *c* were adapted from a previous study[11], which performed time-course microscopic volume measurements on single *S. cerevisiae* cells of S288C background. The parameter values were adjusted to account for the 20 min difference in doubling time, while keeping the same level of noise (measured by standard deviation divided by mean), cell volume, and ratio of *T1'* to *T2* to *T3.* The value of *c* was fixed at 0.25. The average cell doubling time we used in our model was 90 min. Supplementary Table 2 contains a list of the parameter values.

For the gene network module, we fixed rates of RNA transcription and decay[1], protein translation[6,7,13] and degradation[13], as well as basal transcription levels for the GAL network promoters[4] from ranges described in the published literature (Tables S3, S4). As our model applies a fitted scaling parameter to each protein concentration, we do not expect inaccuracies in the fixed parameter values to have significant effects.

We set $\eta = 4$ for the *GAL1* gene and the P_{GAL1}-YFP reporter, and $\eta = 1$ for *GAL3, GAL80* and P_{GAL80}-rtTA, based on the number of Gal4p binding sites on the respective promoters[14,15]. We set $\alpha = 1$ in the GAL network's functional form based on previous work[16]. For P_{TET}-*GAL80*, we set $\gamma = 2$, as rtTA dimerizes, and $\eta = 2$ as the promoter used was TET_{O2}, with two binding sites for rtTA. Finally, we set the global noise level at 10% – that is, the perturbation applied to each rate parameter is sampled from a normal distribution with mean zero and standard deviation equal to 10% of the parameter's value.

E. Stochastic simulations using the model

The model was implemented in custom-written C++ code. Simulations were performed using a modified version[17,18] of the well-established Gillespie algorithm[19].

Cell populations were simulated for 22 hours, corresponding to the amount of time the cells were grown for experimental measurements. In each simulation, the initial population contains 25,000 cells, and the inducers are introduced at $t = 0$. Inspired by a previous work[20], we performed random samplings every 40 minutes to maintain the population at the same size.

The age of the initial cells at $t = 0$ is sampled from an exponential distribution whose mean is equal to the average doubling time (90 minutes). The initial state of each cell is set using the steady state values computed from the parameter values.

The simulation is repeated for various inducer concentrations. The reporter protein count in each cell is converted to simulated fluorescence measurements using a fitting procedure described in section H, below.

F. Fitting other model parameters

Model parameters not fixed from literature in section D are determined via fitting, in two steps. The GAL-network related parameters were determined by fitting the wild-type GAL network model to measurements performed using a haploid WT strain with a P_{GAL1}-YFP reporter (MA0048). The resulting parameter values are shown in Supplementary Table 5. Then, parameters specific to the synthetic network (mostly TET-related parameters) are determined by fitting the synthetic network model to measurements performed using a haploid strain carrying one copy of the synthetic network (WP115). The resulting parameter values are shown in Supplementary Table 6. The parameter values determined in the wild-type fit (MA0048) are then used unchanged in the second fit (WP115).

Given that we have fixed the value of α at 1 (see section D), the functional form of the GAL network simplifies to:

$$F_{\mathrm{GAL}} = \frac{1}{1 + \left(\frac{S_{80}[\mathrm{Gal80p}]}{1 + S_3 g[\mathrm{Gal3p}] + S_1 g[\mathrm{Gal1p}]}\right)^{\beta}}$$

$$\approx \frac{1}{1 + \left(\frac{S_{80}[\mathrm{Gal80p}]}{S_3 g[\mathrm{Gal3p}] + S_1 g[\mathrm{Gal1p}]}\right)^{\beta}} \qquad \text{when } S_3 g[\mathrm{Gal3p}] + S_1 g[\mathrm{Gal1p}] \gg 1$$

As a result, changing the values of S_{80}, S_1 and S_3 proportionally doesn't affect the value of F_{GAL}. We therefore arbitrarily fixed $S_{80} = 30000$ for the first fitting procedure, and fitted only S_1 and S_3. The other fitted parameters are β (nonlinearity coefficient for Gal80p-Gal4p interaction), f

(maximum promoter active fraction), and r (maximum promoter activation rate), for the *GAL80, GAL3,* and *GAL1* promoters, for a total of nine parameters.

As the rtTA-TET network produced Gal80p from a different promoter (P_{TET}), to account for inaccuracies, we included S_{80} in the second fit, along with f, r, and b' (observed basal expression fraction) for the *TET* promoter, and S_{d} (rtTA's scale of action), for a total of five parameters.

For each fitting, we first tried a wide range of possible parameter value combinations to select the initial parameter values that yield fluorescence distributions similar to those experimentally observed. Then, fitting is performed using the well-known Nelder-Mead algorithm[21-23] implemented in the NLopt library (http://ab-initio.mit.edu/nlopt). The simulation is repeated a number of times (denoted N_{R}) for each set of parameter values. Using the fluorescence fitting procedure described in section H, the result of each simulation run is scored for how well it matches the experimental results. The score of the parameter values is the mean of the scores of the individual simulations. The fitting algorithm was first run with a smaller N_{R} (in the 8~20 range), and then with a larger N_{R} (100 or higher), in both stages until significant improvement in the score can no longer be observed.

G. Predicting the activity of the natural and synthetic networks in diploid cells

We used the same doubling time for haploid cells and diploid cells, and it is known the diploid cells have twice the volume on average as haploid cells. Thus, the volume of diploid cells should grow twice as fast compared to a haploid cell. Accordingly, in the diploid cell simulation, all volume module parameters that represent growth rates were doubled, along with the initial volume and the parameter b. In addition, with the exception of the reporter, diploid cells start with two copies of each gene (doubled to four after S phase), rather than one. The reporter copy number is kept at one – two after S phase. All other parameters, whether fixed from literature or obtained from fitting in haploid cells, were kept the same.

H. Fitting procedure for fluorescence

The goal of this fitting procedure is to convert a set of reporter protein counts in n cells $R = \{R_1, R_2, \ldots, R_n\}$, to simulated fluorescence values. First, a set of background fluorescence values $B = \{B_1, B_2, \ldots, B_n\}$ is generated. Each B_i is sampled from a normal distribution; the mean and standard deviation of the distribution are obtained experimentally by using a population of uninduced wild-type cells ($\mu = 52.7,\ \sigma = 16.4$). Given R, B, and a particular reporter-to-fluorescence conversion factor c, the likelihood function is defined as follows.

The total fluorescence for cell i is $F_i = c\,R_i + B_i$. Then, we compute a histogram of $log_{10}(F_i)$, normalized to total area of 1. We define $\mathrm{pdf}(g) = \max(10^{-4}, H_{a,b})$, where $[a,b)$ is the bin containing $\log_{10}(g)$ and $H_{a,b}$ is the height of the bin $[a,b)$ in the histogram. Then, given the known experimental observations of n cells with fluorescence $E_1, E_2, \ldots, E_n$, the likelihood function is given by $L'(R,B,c) = \prod_{i=1}^{n} \mathrm{pdf}(E_i)$. Since the simulation actually generates one R for every inducer concentration used, we correspondingly generate a B for every R, and actual likelihood function $L(c)$ is the product of the values of $L'(R,B,c)$ for each pair of R and B.

Using the Nelder-Mead algorithm[21-23], we attempt to find the value c that minimizes the value of $-\log(L)$ (and so maximizes the value of L). The resulting maximized value of L is the likelihood, and the corresponding value c is the optimal reporter-to-fluorescence conversion factor. During fitting, the above fluorescence fitting procedure is repeated multiple times for each run of the simulation, and the mean of the minimized values of $-\log(L)$ is used as the score of the run.

I. Derivation of the GAL network's functional form

Consider a gene whose promoter transitions between the OFF and ON states, where the rate at which the OFF→ON transition takes place is affected by the concentrations of the relevant regulatory proteins:

$$\mathrm{OFF} \underset{h}{\overset{h\rho(x_1, x_3, x_4, x_{80})}{\rightleftarrows}} \mathrm{ON}$$

The parameter h above represents the timescale at which transitions take place, while $\rho(x_1, x_3, x_4, x_{80})$ is a function that relates the concentrations of the GAL proteins (x_1, x_3, x_4, x_{80}) to the OFF→ON transition rate. We use a functional form to express the activity of the GAL network as a fraction of the maximum activity, which is derived from the interactions of the network components as follows.

Gal4p is the master transcriptional activator of the network, and expressed constitutively. We represent its interaction with a GAL network promoter as

$$\rho = \left(\frac{x_4^*}{K_4}\right)^{\eta}$$

where K_4 represents the typical concentration scale of the interaction, $\eta > 0$ denotes the effective nonlinearity of the Gal4p-promoter interaction, and x_4^* is the active concentration of Gal4p (the fraction not bound by Gal80p and hence able to activate transcription).

Since *GAL4* is a constitutively expressed gene, we take Gal4p's total concentration (denoted by x_4) to be constant. The maximum possible value of ρ is

$$\rho_{\max} = \left(\frac{x_4}{K_4}\right)^{\eta}$$

Since the amount of free Gal4p (x_4^*) should be a decreasing function of Gal80p concentration and an increasing function of total Gal4p, we modeled the Gal4p-Gal80p interaction with the following equation:

$$x_4^* = \frac{x_4}{1 + \left(\frac{x_{80}^*}{K_{80}}\right)^{\beta}}$$

where x_{80}^* is the concentration of Gal80p proteins that are not bound by active Gal3p or Gal1p, K_{80} is the scaling parameter, and β is the degree of nonlinearity of the Gal4p-Gal80p interaction. Despite that Gal1p has a ~40-fold lower inducer activity[24] compared to Gal3p, we included Gal1p in our model as an additional inducer. Gal3p and Gal1p inhibit Gal80p via the same mechanism. Since the amount of x_{80}^* should be a decreasing function of active Gal3p proteins (x_3^*) and active Gal1p proteins (x_1^*), we used the following equation to model the Gal80p-Gal3p/Gal1p interaction:

$$x_{80}^* = \frac{x_{80}}{1 + \left(\frac{x_3^*}{K_3} + \frac{x_1^*}{K_1}\right)^{\alpha}}$$

where x_3^* is the concentration of active Gal3p proteins, x_1^* is the concentration of active Gal1p proteins, K_3 and K_1 are the scaling parameters, and the parameter α quantifies the nonlinearity of Gal80p-Gal3p/Gal1p interaction. Finally, we assume that Gal3p and Gal1p are activated by galactose in a linear fashion, which is true as long as the amount of galactose is non-saturating:

$$x_3^* = x_3 g$$

$$x_1^* = x_1 g$$

Substitution yields

$$\rho = \left(\frac{\frac{x_4}{K_4}}{1 + \left(\frac{\frac{x_{80}}{K_{80}}}{1 + \left(\frac{x_3 g}{K_3} + \frac{x_1 g}{K_1} \right)^{\alpha}} \right)^{\beta}} \right)^{\eta}$$

Thus, the level of network activity, represented as a fraction of the maximum activity, is

$$F = \frac{\rho}{\rho_{\max}} = \left(\frac{1}{1 + \left(\frac{\frac{x_{80}}{K_{80}}}{1 + \left(\frac{x_3 g}{K_3} + \frac{x_1 g}{K_1} \right)^{\alpha}} \right)^{\beta}} \right)^{\eta}$$

Letting $S_3 = \frac{1}{K_3}$, $S_1 = \frac{1}{K_1}$, $S_{80} = \frac{1}{K_{80}}$, we have

$$F = \frac{\rho}{\rho_{\max}} = \left(\frac{1}{1 + \left(\frac{S_{80} x_{80}}{1 + (S_3 x_3 g + S_1 x_1 g)^{\alpha}} \right)^{\beta}} \right)^{\eta}$$

Unlike the other parameters, the value of η may vary from gene to gene, as it depends on the promoter. We therefore handle it in the stochastic model directly. Removing the exponent produces the functional form we aimed to derive:

$$F_{\mathrm{GAL}} = \frac{1}{1 + \left(\frac{S_{80} x_{80}}{1 + (S_3 x_3 g + S_1 x_1 g)^{\alpha}} \right)^{\beta}}$$

Supplementary References

1. Munchel, S.E., Shultzaberger, R.K., Takizawa, N. & Weis, K. Dynamic profiling of mRNA turnover reveals gene-specific and system-wide regulation of mRNA decay. *Molecular Biology of the Cell* **22**, 2787-2795 (2011).
2. Belle, A., Tanay, A., Bitincka, L., Shamir, R. & O'Shea, E.K. Quantification of protein half-lives in the budding yeast proteome. *Proceedings of the National Academy of Sciences of the United States of America* **103**, 13004-13009 (2006).
3. Zacharioudakis, I., Gligoris, T. & Tzamarias, D. A Yeast Catabolic Enzyme Controls Transcriptional Memory. *Current Biology* **17**, 2041-2046 (2007).
4. Hsu, C. *et al.* Stochastic signalling rewires the interaction map of a multiple feedback network during yeast evolution. *Nat Commun* **3**, 682 (2012).
5. Timson, D.J., Ross, H.C. & Reece, R.J. Gal3p and Gal1p interact with the transcriptional repressor Gal80p to form a complex of 1:1 stoichiometry. *Biochemical Journal* **363**, 515-520 (2002).
6. Ghaemmaghami, S. *et al.* Global analysis of protein expression in yeast. *Nature* **425**, 737-741 (2003).
7. To, T.-L. & Maheshri, N. Noise Can Induce Bimodality in Positive Transcriptional Feedback Loops Without Bistability. *Science* **327**, 1142-1145 (2010).
8. Urlinger, S. *et al.* Exploring the sequence space for tetracycline-dependent transcriptional activators: Novel mutations yield expanded range and sensitivity. *Proceedings of the National Academy of Sciences of the United States of America* **97**, 7963-7968 (2000).
9. Nevozhay, D., Adams, R.M., van Itallie, E., Bennett, M.R. & Balázsi, G. Mapping the environmental fitness landscape of a synthetic gene circuit. *PLoS Computational Biology* **8**(2012).
10. Garí, E., Piedrafita, L., Aldea, M. & Herrero, E. A Set of Vectors with a Tetracycline-Regulatable Promoter System for Modulated Gene Expression in Saccharomyces cerevisiae. *Yeast* **13**, 837-848 (1997).
11. Ferrezuelo, F. *et al.* The critical size is set at a single-cell level by growth rate to attain homeostasis and adaptation. *Nat Commun* **3**, 1012 (2012).
12. Alon, U. *An introduction to systems biology: design principles of biological circuits*, (Chapman & Hall, Boca Raton, FL, 2007).
13. Belle, A., Tanay, A., Bitincka, L., Shamir, R. & O’Shea, E.K. Quantification of protein half-lives in the budding yeast proteome. *Proceedings of the National Academy of Sciences* **103**, 13004-13009 (2006).
14. Kellis, M., Patterson, N., Endrizzi, M., Birren, B. & Lander, E.S. Sequencing and comparison of yeast species to identify genes and regulatory elements. *Nature* **423**, 241-254 (2003).
15. Peng, W., Liu, P., Xue, Y. & Acar, M. Evolution of gene network activity by tuning the strength of negative-feedback regulation. *Nat Commun* **6**(2015).
16. Acar, M., Pando, B.F., Arnold, F.H., Elowitz, M.B. & van Oudenaarden, A. A General Mechanism for Network-Dosage Compensation in Gene Circuits. *Science* **329**, 1656-1660 (2010).
17. Gillespie, D.T. Approximate accelerated stochastic simulation of chemically reacting systems. *Journal of Chemical Physics* **115**, 1716-1733 (2001).
18. Tian, T. & Burrage, K. Binomial leap methods for simulating stochastic chemical kinetics. *Journal of Chemical Physics* **121**, 10356-10364 (2004).
19. Gillespie, D.T. Exact stochastic simulation of coupled chemical reactions. *The Journal of Physical Chemistry* **81**, 2340-2361 (1977).

20. Charlebois, D.A., Intosalmi, J., Fraser, D. & Kærn, M. An Algorithm for the Stochastic Simulation of Gene Expression and Heterogeneous Population Dynamics. *Communications in Computational Physics* **9**, 89-112 (2011).
21. Box, M.J. A New Method of Constrained Optimization and a Comparison With Other Methods. *The Computer Journal* **8**, 42-52 (1965).
22. Richardson, J.A. & Kuester, J.L. Algorithm 454: the complex method for constrained optimization [E4]. *Commun. ACM* **16**, 487-489 (1973).
23. Nelder, J.A. & Mead, R. A Simplex Method for Function Minimization. *The Computer Journal* **7**, 308-313 (1965).
24. Platt, A. & Reece, R.J. The yeast galactose genetic switch is mediated by the formation of a Gal4p-Gal80p-Gal3p complex. *EMBO Journal* **17**, 4086-4091 (1998).
